# Macrophage-derived netrin-1 promotes abdominal aortic aneurysm formation by activating MMP3 in vascular smooth muscle cells

**DOI:** 10.1038/s41467-018-07495-1

**Published:** 2018-11-27

**Authors:** Tarik Hadi, Ludovic Boytard, Michele Silvestro, Dornazsadat Alebrahim, Samson Jacob, Jordyn Feinstein, Krista Barone, Wes Spiro, Susan Hutchison, Russell Simon, Debra Rateri, Florence Pinet, David Fenyo, Mark Adelman, Kathryn J. Moore, Holger K. Eltzschig, Alan Daugherty, Bhama Ramkhelawon

**Affiliations:** 1 Division of Vascular Surgery, Department of Surgery, New York University Medical Center, New York, NY 10016 USA; 2 Department of Biochemistry and Molecular Pharmacology, New York University School of Medicine, New York, NY 10016 USA; 3 Leon H. Charney Division of Cardiology, Department of Medicine, New York University School of Medicine, New York, NY 10016 USA; 4 Department of Physiology and Saha Cardiovascular Research Center, University of Kentucky, Lexington, KY 40506 USA; 5 University of Lille, Inserm U1167, Institut Pasteur de Lille, 59019 Lille, France; 6 Department of Anesthesiology, McGovern Medical School, University of Texas Health Science Center at Houston, Houston, TX 77030 USA; 7 Department of Cell Biology, New York University Medical Center, New York, NY 10016 USA

## Abstract

Abdominal aortic aneurysms (AAA) are characterized by extensive extracellular matrix (ECM) fragmentation and inflammation. However, the mechanisms by which these events are coupled thereby fueling focal vascular damage are undefined. Here we report through single-cell RNA-sequencing of diseased aorta that the neuronal guidance cue netrin-1 can act at the interface of macrophage-driven injury and ECM degradation. Netrin-1 expression peaks in human and murine aneurysmal macrophages. Targeted deletion of netrin-1 in macrophages protects mice from developing AAA. Through its receptor neogenin-1, netrin-1 induces a robust intracellular calcium flux necessary for the transcriptional regulation and persistent catalytic activation of matrix metalloproteinase-3 (MMP3) by vascular smooth muscle cells. Deficiency in MMP3 reduces ECM damage and the susceptibility of mice to develop AAA. Here, we establish netrin-1 as a major signal that mediates the dynamic crosstalk between inflammation and chronic erosion of the ECM in AAA.

## Abstract (web summary)

Abdominal aortic aneurysms (AAA) are characterized by extensive extracellular matrix degradation. Here Hadi et al. identify a netrin-1/neogenin-based crosstalk between macrophages and vascular smooth muscle cells (VSMCs), leading to the secretion of the matrix metalloproteinase MMP-3 by VSMCs and subsequent matrix degradation in AAA lesions.

## Introduction

Abdominal aortic aneurysms (AAA) are distinguished by the progressive structural impairment of the abdominal aorta due to extensive vascular injury that manifests as focal arterial enlargement^1^. Because AAA is generally asymptomatic^2^, it is likely that reported prevalence of up to 8% in elderly men and ~13,000 annual mortality attributed to AAA rupture are underestimated^3^. To prevent life-threatening rupture of the weakened vessels, surgical intervention is still the mainstay treatment of this complex multifactorial disease^4^. There is currently an unmet need to ameliorate surgical approaches or to develop therapies that delay surgery in order to improve clinical management of individuals with AAA.

Cumulative efforts to understand the mechanisms that contribute to the local trauma associated with AAA have consistently highlighted the activation of the immune response in the pathological vascular wall^5–7^. Recently, microarray-based gene expression studies have illuminated an overrepresentation of pathways involved in inflammatory responses^8–10^, establishing further evidence that AAA is an immunologic disease with dominant roles described for activated monocytes/macrophages subsets^6^. The mechanisms by which monocyte-derived macrophages are channeled to the AAA location have been well defined and multiple players including C–X–C motif receptor 4 (CXCR4)^11^, chemoattractant protein-1 receptor (CCR2)^12^ and its ligand chemokine (C–C motif) ligand 2 (CCL2)^13^ have been shown to play pivotal roles in directing these steps. The coordinated action of CCL2 and interleukin-6 (IL-6)^14^ also nurtured the supply of monocyte-derived macrophages to the vascular wall in apolipoprotein E deficient mice (*ApoE*^*−/−*^) exposed to continuous infusion of angiotensin II (Ang II), a prototypical AAA model^15^. However, why and how monocyte/macrophage accrual in the arterial wall impacts the focal distribution of AAA and the specific role of these cells in AAA is still unclear.

In healthy aorta characterized by a paucity of active inflammatory cell invasion, the consolidated ECM plays an instrumental role to limit aortic dysfunction^16^. ECM provides physical scaffold for cells constituent of the aorta and it is therefore constantly remodeled to maintain the integrity of the vascular framework^16^. In AAA, the chronic catabolic activity of ECM-degrading enzymes matrix metalloproteinases (MMP) has consistently been demonstrated to lethally compromise vessel remodeling and precipitate to aortic rupture^17,18^. The presence of macrophages is often associated with MMP activity in AAA suggesting a relationship between macrophages and ECM metabolism. However, the mechanisms that regulate such interactions remained unclear so far. In the quest to identify signals that mediate this crosstalk, we hypothesized that cues displaying immunoregulatory functions could intercept in this process.

Work from our group has identified netrin-1, a neuronal guidance cue with unique laminin-like structure, as a key chemorepulsive protein that directs leukocyte migration^19–21^. Here, we directly address the role of netrin-1 in bridging macrophage-dependent inflammation and sustained ECM damage in AAA. Using single-cell RNA-sequencing (RNA-seq) of AAA, we identify that pools of activated macrophages express netrin-1. The deficiency of netrin-1 in hematopoietic lineage and in macrophages protects mice from developing AAA compared to wild type (WT) littermates. Netrin-1 modulates calcium mobilization in neighboring vascular smooth muscle cells (VSMCs) via its receptor, neogenin-1. Activation of neogenin-1 by netrin-1 is required for the nuclear translocation of the transcription factor nuclear factor of activated T-cells, cytoplasmic 3 (NFATc3) to potentiate the catalytic activity of matrix metalloproteinase-3 (MMP3). The deletion of MMP3 protects mice from developing AAA. Our data suggest that locally activated macrophages leave a netrin-1 imprint in AAA, thereby fueling MMP3 activation by VSMCs and fostering focal degradation of the ECM that manifests in AAA.

## Results

### Netrin-1 is predominant in murine and human AAA

**Fig. 1:**
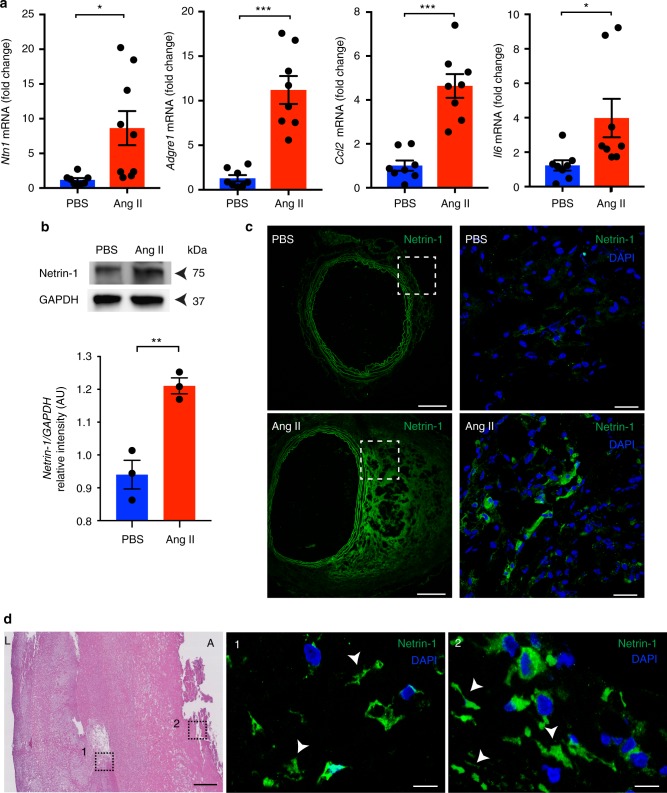
Netrin-1 is highly expressed in human and murine AAA. **a** Quantitative PCR analysis of *Ntn1*, *Adgre1*, *Ccl2, and Il6* mRNA levels isolated from aortas of *ApoE*^*−/−*^ mice exposed to PBS or Ang II for 28 days (*n* = 8/9 mice per group). *P *<* 0.05, ***P < 0.001. **b** Western blot analysis of netrin-1 in aorta extracts normalized to loading control, GAPDH (*n* = 3 mice per group). AU arbitrary units. ** *P* < 0.01. Representative microscopy staining of netrin-1 and cell nuclei (DAPI, blue) in magnified aortic sections (dashed box) from PBS and Ang II infused *ApoE*^*−/−*^ mice (**c**) (whole aortic section is shown in left top and bottom, scale bar 200 µm, magnified areas on the right, scale bar 20 µm) and human specimens (**d**); Hematoxylin and eosin (H&E; scale bar 500 µm) staining showing magnified sections (1, 2; scale bar 20 µm); arrows indicate acellular localization of netrin-1; L lumen, A adventitia. Unpaired, two-tailed *t*-test was used for statistical analysis. Error bars represent s.e.m

Transmural inflammation is detrimental to ECM integrity in AAA. Important roles of netrin-1 in orchestrating inflammation have been established^20,22–24^. In the quest to identify signals that can coordinate focal vascular injury and sustained inflammation in AAA, we hypothesized that netrin-1 could play a role in this context. We first characterized the level of netrin-1 in diseased sections of the aorta in an established murine AAA modeled through the infusion of Ang II in *ApoE*^*−/−*^ mice^15^. Quantitative RT-PCR revealed that netrin-1 mRNA (*Ntn1*) expression was significantly increased in AAA aortas compared to healthy tissues exposed to PBS. A similar expression pattern of inflammatory markers adhesion G-protein-coupled receptor E1 (*Adgre1*), interleukin 6 (*Il6*) and the C-C motif chemokine 2 (*Ccl2*) was observed in the aortas, consistent with previous reports^25^ (Fig. 1a). Western blot analysis confirmed a marked increase in the expression of netrin-1 within the AAA loci compared to healthy aortas (Fig. 1b). This coincided with immunofluorescence staining of aortic sections that revealed a pronounced pattern for netrin-1 in cellular and acellular structures, both in murine tissues (Fig. 1c) as well as in human sections (Fig. 1d). Collectively, these results suggested that the accumulation of netrin-1 may play a causative role in AAA.

### Netrin-1 deficiency in hematopoietic lineage prevents AAA

**Fig. 2:**
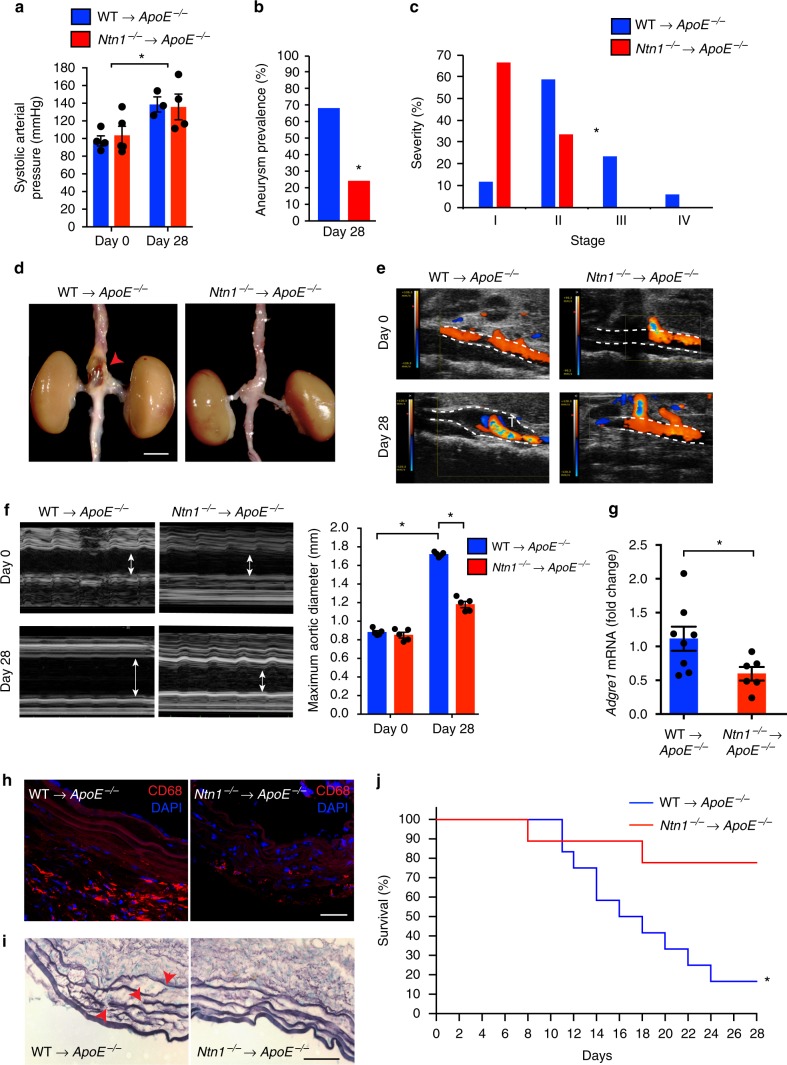
Netrin-1 deficiency in hematopoietic lineage protects against AAA. Arterial pressure measurement (**a**), aneurysm prevalence (**b**), and severity stratification (**c**) of WT→*ApoE*^*−/−*^ and *Ntn1*^*−/−*^→*ApoE*^*−/−*^ fetal liver chimeras exposed to Ang II for 0 or 28 days (*n* = 3–5 (**a**), *n* = 25 (**b**, **c**), *P < 0.05). **d** Representative photomicrograph of aortas at sacrifice. Arrowhead indicates abdominal aneurysm. *n* = 6 for WT→*ApoE*^*−/−*^ and *n* = 8 for *Ntn1*^*−/−*^→*ApoE*^*−/−*^; scale bar 2 mm. Representative color Doppler ultrasound images of abdominal aortas at day 0 and day 28 in color mode (**e**) and in M-mode (**f**) and measurements of maximum diameter at day 0 and 28 (arrows indicate vessel enlargement) *n* = 5; WT→*ApoE*^*−/−*^ and *n* = 5; *Ntn1*^*−/−*^→*ApoE*^*−/−*^. *P < 0.05. **g** mRNA levels of *Adgre1* by quantitative PCR in aortas of WT→*ApoE*^*−/−*^ (*n* = 8) and *Ntn1*^*−/−*^→*ApoE*^*−/−*^ mice (*n* = 6). *P < 0.05. **h** Representative immunofluorescence staining of macrophage marker CD68 (red) and nuclei (DAPI, blue); scale bar 50 µm; *n* = 3. **i** Example of elastin staining (Verhoeff–Van Gieson staining). Red arrowheads indicate points of elastin breaks; scale bar 50 µm; *n* = 3. **j** Kaplan–Meier survival curves as indicated. WT→*ApoE*^*−/−*^, *n* = 9; *Nt1n*^*−/−*^→*ApoE*^*−/−*^*, n* = *12*. *P < 0.05. Unpaired, two-tailed *t*-test was used for statistical analysis in **a**, **c**, and **g**. Two-sided Fisher’s exact test was used to compare variables in **b**. Two-way ANOVA was used in **f**. Statistical significance on Kaplan–Meier curves (**j**) has been assessed with Mantel–Cox test. Data error bars represent s.e.m

To gain insight into the role of netrin-1 in the development of AAA, we investigated the impact of the lack of netrin-1 in AAA. To circumvent the embryonic lethality of netrin-1 deficiency, we used a fetal liver transplant strategy where lethally irradiated recipient *ApoE*^*−/−*^ mice were reconstituted with either *Ntn1*^*−/−*^*ApoE*^*−/−*^ or *Ntn1*^*+/+*^*ApoE*^*−/−*^ day-14 embryonic cells. We therefore generated *Ntn1*^*−/−*^*ApoE*^*−/−*^ (*Ntn1*^*−/−*^→*ApoE*^*−/−*^) or *Ntn1*^*+/+*^*ApoE*^*−/−*^ (WT→*ApoE*^*−/−*^) spontaneously hypercholesterolemic chimera mice that we challenged to Ang II or PBS for 28 days after transplantation recovery. While Ang II infusion increased the blood pressure from day 0 to day 28, no different pro-hypertensive effects were observed between chimeric *Ntn1*^*−/−*^→*ApoE*^*−/−*^ and WT→*ApoE*^*−/−*^ animals (Fig. 2a). Interestingly, although ~70% of the WT→*ApoE*^*−/−*^ animals developed AAA, only ~25% of *Ntn1*^*−/−*^→*ApoE*^*−/−*^ mice showed features of the disease (Fig. 2b). Analysis of the detailed severity of AAA classified by stages, as previously described^26^, showed that *Ntn1*^*−/−*^→*ApoE*^*−/−*^ chimeras were protected from developing complex manifestations of AAA typified by prominent aortic bulging and transmural thrombus in the supra-renal regions (Fig. 2c, d). To closely profile the hemodynamic features and non-invasively monitor the progression of aortic enlargement, we performed color Doppler ultrasound imaging weekly. Consistent with the incidence of AAA, turbulent flow patterns illustrated by dual-color blood flow profiles and aliasing effects were captured longitudinally in WT→*ApoE*^*−/−*^ mice in contrast to the laminar flows acquired in the supra-renal region of *Ntn1*^*−/−*^→*ApoE*^*−/−*^ mice treated Ang II (Fig. 2e). These prototypical features manifest in the human pathology and were recapitulated in our present chimeric murine models. Notably, vessel dilatation was markedly increased in WT→*ApoE*^*−/−*^ mice exposed to Ang II compared to PBS treated mice, however, the aortic diameter was reduced in *Ntn1*^*−/−*^→*ApoE*^*−/−*^ mice treated with Ang II (Fig. 2f). These data suggested that the absence of netrin-1 in the hematopoietic compartment could protect against the development of AAA. Since inflammation and ECM degradation are key hallmarks of AAA, we therefore assessed macrophage infiltration and elastin damage. The accrual of macrophages characterized by mRNA abundance of *Adgre1* (Fig. 2g) and immunostaining directed against CD68 (Fig. 2h) was significantly reduced in *Ntn1*^*−/−*^→*ApoE*^*−/−*^ as opposed to WT→*ApoE*^*−/−*^ aortas that were prone to AAA. Extensive elastin damage depicted by focal breaks and fiber thinning was consistently observed across the circumference of the aortic sections of the WT→*ApoE*^*−/−*^ mice whereas those examined from *Ntn1*^*−/−*^→*ApoE*^*−/−*^ sections were in opposite mirror to the latter findings (Fig. 2i). These exciting key observations predicted that the expression of netrin-1 could contribute to elastin damage and precipitate to vessel rupture. To test this hypothesis, we subjected a subgroup of older mice (~11 months) to high dose of Ang II as previously described^14^. While mortality peaked to 80% in the WT→*ApoE*^*−/−*^ group, 80% of the *Ntn1*^*−/−*^→*ApoE*^*−/−*^ mice survived after 28 days of treatment (Fig. 2j). Altogether, these findings clearly indicated a seminal role for hematopoietic-derived netrin-1 in steering the destruction of the aortic wall in AAA.

### Unique signature of netrin-1 in aortic macrophage subset

**Fig. 3:**
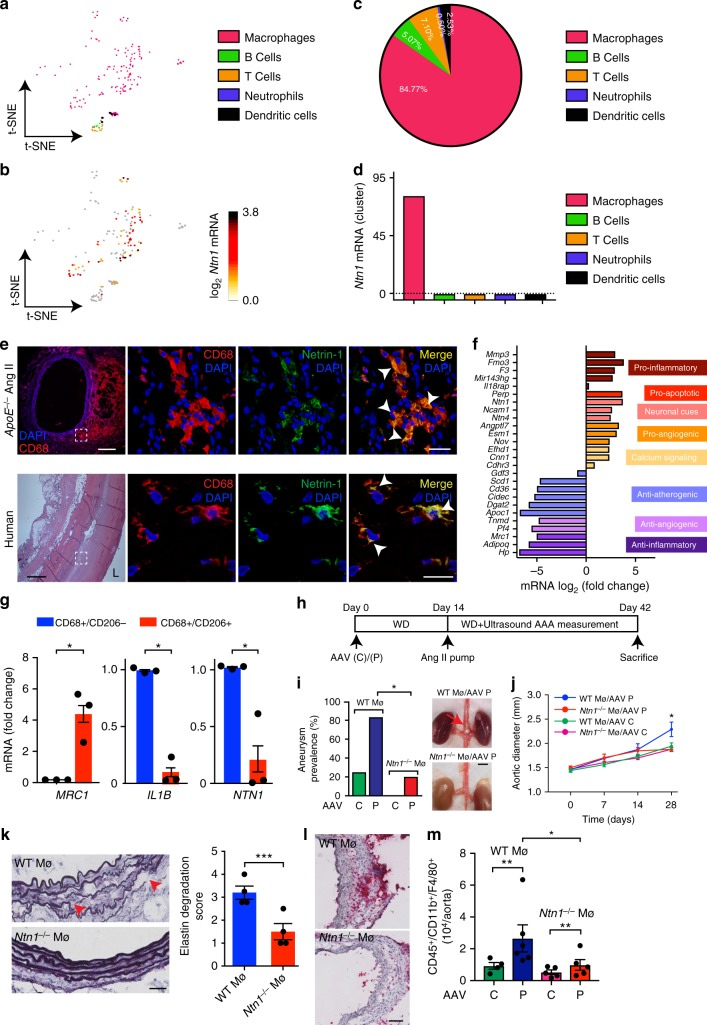
Netrin-1 deletion in macrophages inhibits AAA development. *t*-distributed stochastic neighbor embedding (t-SNE) plot of single-cell RNA-sequencing of diseased aortas (*n* = 3, pooled). Leukocyte clusters are color coded as shown (**a**), *Ntn1* mRNA distribution (log_2_) within the clusters (**b**). **c** Pie chart showing percentage of *Ntn1* mRNA per immune cell population. **d** Quantification of *Ntn1* mRNA abundance per cell cluster. **e** Representative immunofluorescence staining of CD68 and netrin-1 showing colocalization (merge; arrows) in aortic sections of Ang II mice (upper panels; scale bar 20 µm, except left panel, scale bar 200 µm) and human aneurysmal tissue (lower panels; scale bar 20 µm, except left panel, scale bar 500 µm). Dashed boxes indicate magnification sites displayed in right panels. Representative H&E staining of human section is shown. L lumen. *n* = 3. **f** Transcripts expression in *Ntn1* positive (right) and *Ntn1* negative (left) macrophages. Genes are clustered according to biological process. **g** *MRC1, IL1B*, *and NTN1* mRNA expression in human aneurysmal laser micro-dissected segments enriched in CD68^+^/CD206^+^ (*n* = 3) or CD68^+^/CD206^−^ (*n* = 3) macrophages. Unpaired, two-tailed *t*-test, *P < 0.05. **h** Schematic representation of PCSK9 (P) or control (C) adeno-associated virus vector (AAV) and Ang II treatments in C57BL/6J WT (WTMø) and *Ntn1*^fl/fl^/LysM-Cre^+/−^ (*Ntn1*^*−/−*^Mø) mice. WD western diet. Aneurysm prevalence with representative photomicrographs of aortas (**i**) (*n* = 15, WTMø/AAV P, n = 5 *Ntn1*^*−/−*^ Mø/AAV P. Two-sided Fisher’s exact test; *P < 0.05. Red arrow indicates AAA) and aortic diameter (**j**) of WTMø and *Ntn1*^*−/−*^Mø mice exposed to C or P AAV. (*n* = 4 mice; WT and *Ntn1*^*−/−*^ Mø/AAV C), (*n* = 5; WT and *Ntn1*^*−/−*^ Mø/AAV P). Two-way ANOVA, *P < 0.05. **k** Microscopy images of elastin content (Verhoeff–Van Gieson staining) and elastin degradation score. Red arrowheads show elastin breaks. *n* = 3; scale bar 50 µm; unpaired, two-tailed *t*-test, ***P < 0.001. **l** Representative aortic sections stained for CD68 macrophages; scale bar 50 µm. **m** Quantification of macrophages (CD45^+^/CD11b^+^/F4/80^+^) by flow cytometry. *n* = 4; WT and *Ntn1*^*−/−*^ Mø/AAV C), (*n* = 6; WT and *Ntn1*^*−/−*^ Mø/AAV P). Unpaired, two-tailed *t*-test, **P < 0.01. Error bars represent s.e.m

In the aim to define which cellular entity among the hematopoietic lineage was responsible for the expression of netrin-1, we profiled the transcriptome landscape of murine AAA through single-cell RNA-seq. Using *t*-distributed stochastic neighbor embedding (*t*-SNE) nonlinear dimension-reduction method, distinct immune sub-populations were identified based on their variability in genetic distribution patterns in the diseased aorta (Fig. 3a). Screening for *Ntn1* transcript within the clusters identified a unique signature for *Ntn1* among the heterogeneous immune cells (Fig. 3b). Interestingly, amongst these hematopoietic clusters, ~83% of the cells that expressed *Ntn1* were macrophages (Cd11b^+^/Cd68^+^/Adgre1^+^) while only ~7% of T cells (*Cd3e*^*+*^), ~5% of B cells (*Cd19*^*+*^), ~0.5% of neutrophils (*Ly6g*^*+*^*)*, and ~3% of dendritic cells (*Itgax*^*+*^) were detected as *Ntn1* positive (Fig. 3c). This was consistent with the mean level of *Ntn1* transcript abundance in each cluster which distinctly culminated in macrophages (Fig. 3d). Double immunofluorescence staining of diseased murine AAA sections confirmed the marked presence of netrin-1 protein in CD68 positive macrophages (Fig. 3e, top) as compared to PBS-infused mice (Supplementary Figure 1a). Similar results were recapitulated in human sections (Fig. 3e, bottom), further validating the data obtained by the single-cell RNA-seq and emphasizing on the translational impact of macrophage-dependent expression of netrin-1 in AAA. Transcriptomic analysis of the macrophage cluster using the Loupe Cell Browser software expanded the set of genes that best discriminate the pools of *Ntn1* positive macrophages within the inflamed aortic vessel wall. Of note, we found that *Ntn1* enriched macrophages were those that highly expressed pro-inflammatory markers and pro-angiogenic markers, while macrophages that harbored lower levels of *Ntn1* exhibited high anti-inflammatory macrophage mannose receptor 1 mRNA (*Mrc1*) and anti-atherogenic repertoire of genes consistent with previous reports^20,21^ (Fig. 3f). Since macrophage mannose receptor 1 (CD206) is a recognized prototypical anti-inflammatory marker of tissue macrophages, we performed laser capture microdissection of human AAA sections to selectively isolate either CD68^+^CD206^−^or CD68^+^CD206^+^ segments of the tissue, as previously described^27^. As shown in Fig. 3g, *MRC1* transcript was enriched in CD68^+^CD206^+^ segments captured from three patients, while pro-inflammatory marker *IL1B* was only expressed in CD68^+^CD206^−^ samples, confirming the purity of the captured aortic portions. Interestingly, *NTN1* mRNA was increased in CD68^+^CD206^−^ clusters suggesting that, in vivo, macrophages exhibiting pro-inflammatory phenotypes compatible with tissue damage, were those where the expression of netrin-1 peaked, indicative of a causative role of macrophage-derived netrin-1 in AAA.

### Netrin-1 deletion in macrophages inhibits AAA development

To directly address the specific role of macrophage-derived netrin-1 in AAA, mice harboring *Ntn1* gene flanked by lox-P sequences were bred with mice expressing the Cre recombinase in mature macrophage lineage within the lysozyme C-2 gene *Lyz2* (LysMcre) to generate *Ntn1*^flox/flox^LysMcre^+/−^ (*Ntn1*^*−/−*^Mø) and *Ntn1*^flox/flox^LysMcre^-/-^ Nnt1^l^^−/−^ (WTMø) control mice. No phenotypic defects were observed  in these mice and the reduced expression of netrin-1 in monocytes/macrophages was verified (Supplementary Figure 1b). Since the incidence of AAA is low in the absence of hypercholesterolemia^28^ and in order to increase the susceptibility of normocholesterolemic *Ntn1*^*−/−*^Mø and WTMø C57BL/6 mice to AAA, we injected the mice with a single dose of an adeno-associated virus (AAV) vector expressing the D374Y gain-of-function proprotein convertase subtilisin/kexin type 9 (PCSK9) that resulted in sustained hypercholesterolemia, as described previously^29^. As shown in the schematic in Fig. 3h, following either control or PCSK9 AAV injection, mice were exposed to a western diet (WD) for 2 weeks before Ang II pump was implanted for an additional 4 weeks, as previously described^30^. Combined exposure of Ang II and PCSK9 AAV increased the prevalence of AAA in WT Mø mice by a 4-fold factor compared to mice exposed to control AAV and Ang II. Interestingly, *Ntn1*^*−/−*^ Mø mice were less susceptible to AAA when challenged to control AAV in the presence of Ang II and only 20% that received PCSK9 AAV in conjunction with Ang II developed features of AAA, as depicted in the macroscopic images of the renal region of the aorta (Fig. 3i). Aortic diameter revealed that the aorta of WT Mø mice gradually expanded over time and peaked at 28 days after treatment (Fig. 3j). The lack of netrin-1 in macrophages protected mice from aortic enlargement similar to healthy animals, consistently with preserved integrity of elastin fibers (Fig. 3k) and reduced accumulation of inflammatory CD68 positive macrophages identified by immunohistochemistry of serial aortic sections (Fig. 3l). Flow cytometry of diseased aortic tissue confirmed the reduced accumulation of CD45^+^/CD11b^+^/F4/80^+^ macrophages in the vascular wall of *Ntn1*^*−/−*^Mø mice compared to WTMø (Fig. 3m). These results were in line with the protective phenotype observed with *Ntn1*^*−/−*^→*ApoE*^*−/−*^ chimeras and clearly suggest that the absence of netrin-1 in macrophages reduces the susceptibility to develop AAA.

### Netrin-1 regulates the activity of MMP3 in the vascular wall

**Fig. 4:**
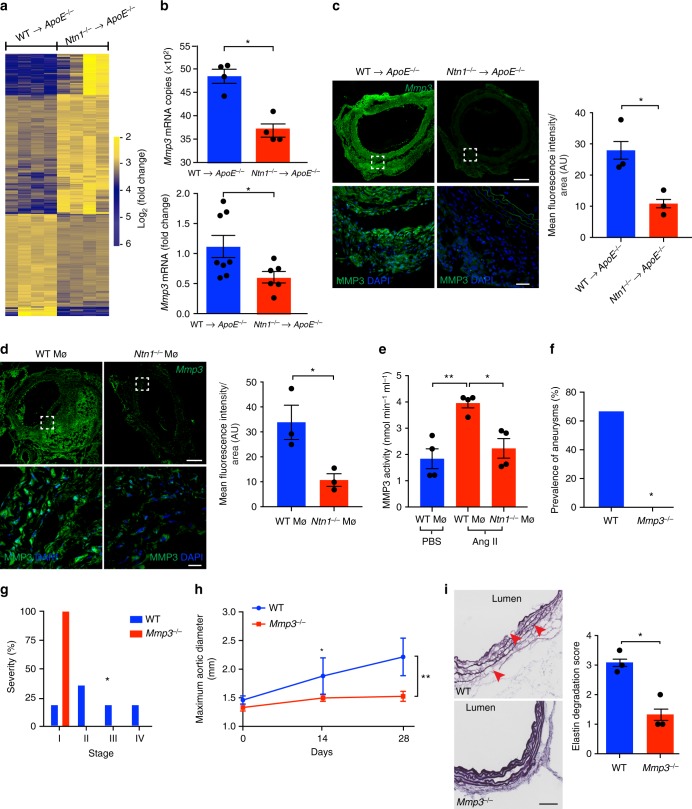
Netrin-1 regulates MMP3 activity in AAA. **a** Heatmap representation of transcriptomic profiling of WT→*ApoE*^*−/−*^ and *Ntn1*^*−/−*^→*ApoE*^*−/−*^ aorta by RNA-sequencing (RNA-seq). *n* = 4 per group. **b** *Mmp3* mRNA copy numbers identified by RNA-seq (*n* = 4 per group) and quantitative PCR analysis of *Mmp3* transcript in aortas (*n* = 8; WT→*ApoE*^*−/−*^, *n* = 6; *Ntn1*^*−/−*^→*ApoE*^*−/−*^). *P < 0.05. Representative immunofluorescence staining and quantification of MMP3 in aortic sections of WT→*ApoE*^*−/−*^ and *Ntn1*^*−/−*^→*ApoE*^*−/−*^ (**c**) and WTMø and *Ntn1*^*−/−*^Mø (**d**) (*n* = 3 per group); upper panels, scale bar 200 µm. Bottom panels (scale bar 20 µm) show magnified region of dashed box. *P < 0.05 (**e**) MMP3 catalytic activity assay in aortic lysates of PBS infused WT, WTMø and *Ntn1*^*−/−*^Mø (*n* = 4 per group) mice treated with Ang II. *P < 0.05, **P < 0.01. Aneurysm prevalence (**f**), severity stratification (**g**), and aortic enlargement (**h**) of WT (*n* = 6) and MMP3 whole body-deficient mice (*Mmp3*^*−/−*^) (*n* = 7) exposed to Ang II. *P < 0.05, **P < 0.01. **i** Representative image of elastin (Verhoeff–Van Gieson staining) and degradation scored in the aortic media. Red arrowheads indicate elastin rupture. Scale bar 100 µm; *n* = 3 per group. *P < 0.05. Unpaired, two-tailed *t*-test was used for statistical analysis in **b**–**e**, **g**, and **i**. Two-sided Fisher’s exact test was used to compare variables in **f**. Two-way ANOVA was used in **h**. Data error bars represent s.e.m

Since transcriptional mapping can provide key mechanistic insights in an unbiased manner, we performed RNA-seq of aortas isolated from WT→*ApoE*^*−/−*^ and *Ntn1*^*−/−*^→*ApoE*^*−/−*^ mice exposed to Ang II to uncover the mechanisms underlying the undefined role netrin-1 in AAA. Through this sensitive RNA profiling of four individual aortas in each experimental group, we generated a library of distinct transcriptome expression patterns as revealed by the volcano plot (Supplementary Figure 2a). Utilizing an adjusted nominal *P* value *<* 0.05, we designed a heatmap representation that captured a large range of expression levels of transcriptome imprints in AAA-prone compared to AAA-protected groups (Fig. 4a). The consistency in the clustering suggested a homogeneous genomic activity with limited background signal and high levels of reproducibility across the biological replicates in each group. Pathway analysis of this library tracked significant and synchronized networks regulated by netrin-1 in the vascular wall. Established functions of netrin-1 including axon development, apoptotic process and cytoskeleton-dependent intracellular transport^19–21,31^ were highlighted through software analysis (Supplementary Figure 2b) confirming the robustness of the data. Compellingly,  Mmp3, a key extracellular matrix degrading enzyme catalyzing a broad range of substrates with accessory inflammatory properties^32,33^, was amongst the most significantly downregulated genes in the aortas of *Ntn1*^*−/−*^→*ApoE*^*−/−*^ mice (Fig. 4b-top). RT-PCR of a larger experimental group validated the marked differential expression of *Mmp3* transcripts in diseased aortas (Fig. 4b-bottom). We further established the downregulation of MMP3 protein expression in *Ntn1*^*−/−*^→*ApoE*^*−/−*^ aortic segments by immunofluorescence, that revealed a significantly reduced expression of MMP3 per section analyzed (Fig. 4c), in accordance with the RNA-seq and RT-PCR results. Importantly, immunofluorescence staining showed a strong decrease of MMP3 in aortic sections isolated from *Ntn1*^*−/−*^Mø mice, that exhibited a dampened susceptibility to develop AAA similar to *Ntn1*^*−/−*^→*ApoE*^*−/−*^ chimeric mice, compared to WT Mø aortic sections (Fig. 4d). This was paralleled by decreased MMP3 catalytic function in aorta isolated from *Ntn1*^*−/−*^Mø as opposed to WTMø mice exposed to Ang II (Fig. 4e). These results clearly evidenced that netrin-1 could fuel the expression of MMP3 with potent ECM degrading capacity in the setting of AAA.

### MMP3 activity promotes AAA

To investigate the direct role of MMP3 in AAA, we challenged MMP3 deficient mice (*Mmp3*^*−/−*^) to Ang II infusion prior to PCSK9 AAV injection. Blood pressure was significantly increased in mice after 28 days of treatment compared to the baseline measurements. No difference between *Mmp3*^*−/−*^ and WT mice blood pressure was observed at time of sacrifice (Supplementary Figure 2c).  Notably, MMP3 deficiency substantially reduced both the incidence (Fig. 4f), the severity (Fig. 4g) of AAA and aortic dilation (Fig. 4h) in comparison to WT mice (Supplementary Figure 2d). Accordingly, extensive elastin degradation was apparent in the vessel wall of WT mice with active MMP3, while MMP3 deficiency maintained the integrity of elastin fibers characterized by a reduction in elastin degradation score (Fig. 4i). Altogether, our data demonstrate a distinct aggravating role of MMP3 in AAA.

### Macrophage-deficient MMP3 fail to rescue AAA

**Fig. 5:**
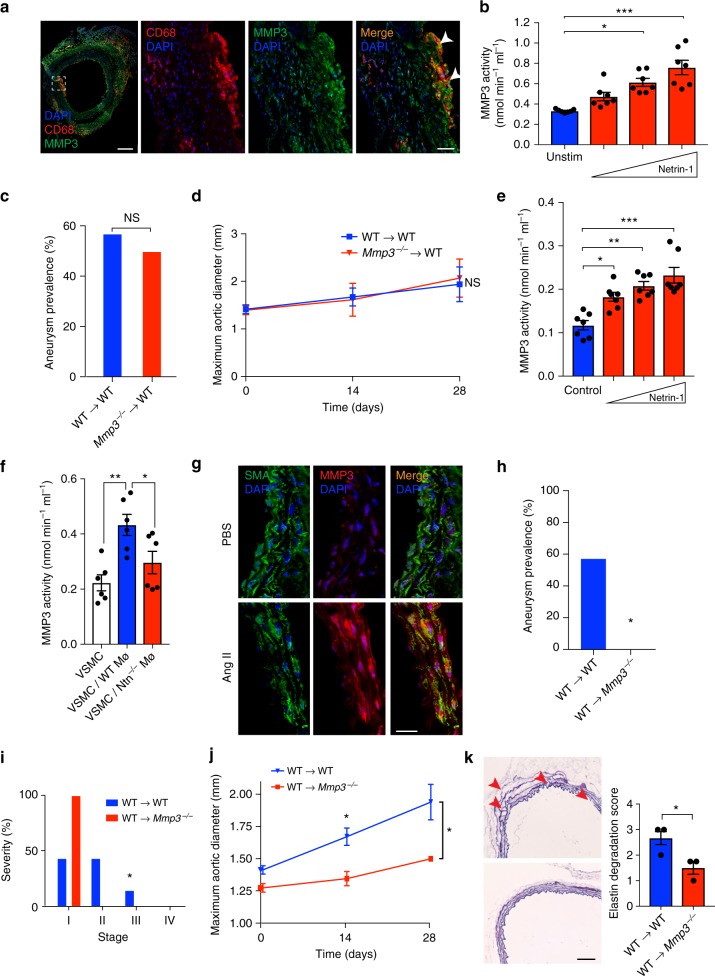
MMP3 contributes to AAA. **a** Representative immunofluorescence staining of CD68, MMP3 and their colocalization (merge, yellow) in *ApoE*^*−/−*^ mice aneurysmal aortic sections. Dashed box represents magnified area. Nuclei stained with DAPI (blue); scale bar 50 µm, except for left panel, scale bar 200 µm. **b** Quantification of MMP3 enzymatic activity in BMDMs stimulated with recombinant netrin-1 (left to right: 0.625, 1.25, and 2.5 µg/ml) for 6 h. *n* = 7. *P < 0.05, ***P < 0.001. Aneurysm prevalence (**c**) and aortic diameter (**d**) of bone marrow transplant WT→WT and *Mmp3*^*−/−*^→WT chimeras. MMP3 enzymatic activity in VSMCs stimulated with recombinant netrin-1 (**e**) (left to right: 0.625, 1.25, and 2.5 µg/ml, *n* = 7 per group) or cultured in presence or absence of WTMø (blue) or *Ntn1*^*−/−*^Mø (red) (**f**) (*n* = 6 per group). *P < 0.05, **P < 0.01. **g** Immunofluorescence staining of MMP3 (red) and alpha-smooth muscle actin (SMA, green) in aortic sections of *ApoE*^*−/−*^ mice infused with PBS or Ang II. Colocalization is shown in merge (yellow). Nuclei stained with DAPI (blue). Scale bar 50 µm. Aneurysm prevalence (**h**), severity stratification (**i**) (WT→WT, *n* = 13 and WT→*Mmp3*^*−/−*^, *n* = 4. *P < 0.05) and aortic diameter (**j**) of WT→WT (*n* = 7) and WT→*Mmp3*^*−/−*^ (*n* = 4) chimeras. *P < 0.05. **k** Elastin content (Verhoeff–Van Gieson staining) and degradation score. Red arrowheads indicate points of elastin rupture. Scale bar 100 µm. *n* = 3 per group. *P < 0.05. One-way ANOVA was used to assess statistical significance in **b** and **e**. Two-sided Fisher’s exact test was used to compare variables in **c** and **h**. Data in **d** and **j** were analyzed by two-way ANOVA. Unpaired, two-tailed *t*-test was used for statistical analysis in **f**, **i**, and **k**. Error bars represent s.e.m

We further characterized which cellular subsets were responsible for MMP3-dependent AAA development. Because macrophages are important sources of MMP3 in atherosclerotic lesions^34^, we performed immunofluorescence to detect whether MMP3 was expressed by macrophages harbored in AAA. MMP3 colocalized with CD68 positive macrophages recruited in the diseased aorta (Fig. 5a). We therefore assessed whether netrin-1 could regulate MMP3 dynamics in macrophages. Recombinant netrin-1 indeed dose-dependently increased MMP3 enzymatic activity in bone marrow-derived macrophages (BMDMs) (Fig. 5b). To assess the role of macrophage-derived MMP3 in vivo, we reconstituted lethally irradiated WT recipient mice with either WT or *Mmp3*^*−/−*^ bone marrow cells to generate WT→WT and *Mmp3*^*−/−*^→WT bone-marrow chimeras. After recovery, mice were exposed to gain-of-function PCSK9 AAV prior to Ang II infusion (Supplementary Figure 3a). Unexpectedly, we did not observe any difference neither in the incidence nor in the severity of AAA between WT→WT and *Mmp3*^*−/−*^→WT groups (Fig. 5c, Supplementary Figure 3b). Similarly, although maximum vessel diameter increased gradually weekly, no difference in vessel enlargement was detected between WT→WT and *Mmp3*^*−/−*^→WT groups (Fig. 5d). These data suggested that although netrin-1 could induce the activity of MMP3 in macrophages in vitro, the deletion of MMP3 in the myeloid lineage was insufficient to confer protection against AAA in vivo.

### Vascular smooth muscle cell-derived MMP3 aggravates AAA

*Mmp3*^*−/−*^ mice were protected against AAA. Conversely, mice with specific MMP3 deficiency in myeloid cells developed AAA. We therefore speculated that MMP3 expression by resident vascular cells within the arterial wall might contribute to the development of AAA. Stimulation of endothelial cells or fibroblasts with increasing concentrations of recombinant netrin-1 did not increase the activity of MMP3 (supplementary Figure 3e, f). However, exposure of murine primary VSMCs to recombinant netrin-1 significantly induced the enzymatic activity of MMP3 in a dose-dependent way (Fig. 5e). Notably, the co-culture of activated WT macrophages with VSMCs in transwell assays promoted MMP3 activity in VSMCs. Similar assays in the presence of *Ntn1*^*−/−*^ Mø abrogated MMP3 activity in VSMCs (Fig. 5f). These data suggested that macrophage-derived netrin-1 could induce MMP3 activity in VSMCs. Interestingly, immunofluorescence of AAA sections revealed a distinct MMP3 expression pattern in VSMCs (Fig. 5g). We henceforth investigated whether VSMCs-derived MMP3 could contribute to the pathogenesis of AAA. Since no MMP3 conditional mice is commercially available, we reconstituted MMP3^−/−^ recipient mice with bone marrow cells isolated from WT mice to generate WT→*Mmp3*^*−/−*^ or WT→WT chimeras in order to delete MMP3 only in cells from non-hematopoietic origin, including VSMCs. After bone marrow transplantation recovery period, WT→*Mmp3*^*−/−*^ and control WT→WT mice were exposed to gain-of-function PCSK9 AAV prior to Ang II infusion. The absence of MMP3 in stromal compartment completely protected WT→*Mmp3*^*−/−*^ animals from developing AAA compared to WT→WT mice (Fig. 5h) albeit no difference in systemic blood pressure (Supplementary Figure 3c). WT→*Mmp3*^*−/−*^ exhibited mild stages of the disease in agreement with reduced aortic enlargement (Fig. 5i) (Supplementary Figure 3d). The aortic diameter was also significantly reduced in WT→*Mmp3*^*−/−*^mice as opposed to WT→WT littermates (Fig. 5j). Consistent with these observations, the deletion of MMP3 in the stromal compartment preserved healthy elastin fibers (Fig. 5k). Altogether, our data point out to the instrumental role of macrophage-derived netrin-1 in driving MMP3 catalytic activity in VSMCs.

### Netrin-1 fine tunes calcium influx to activate MMP3 in VSMCs

**Fig. 6:**
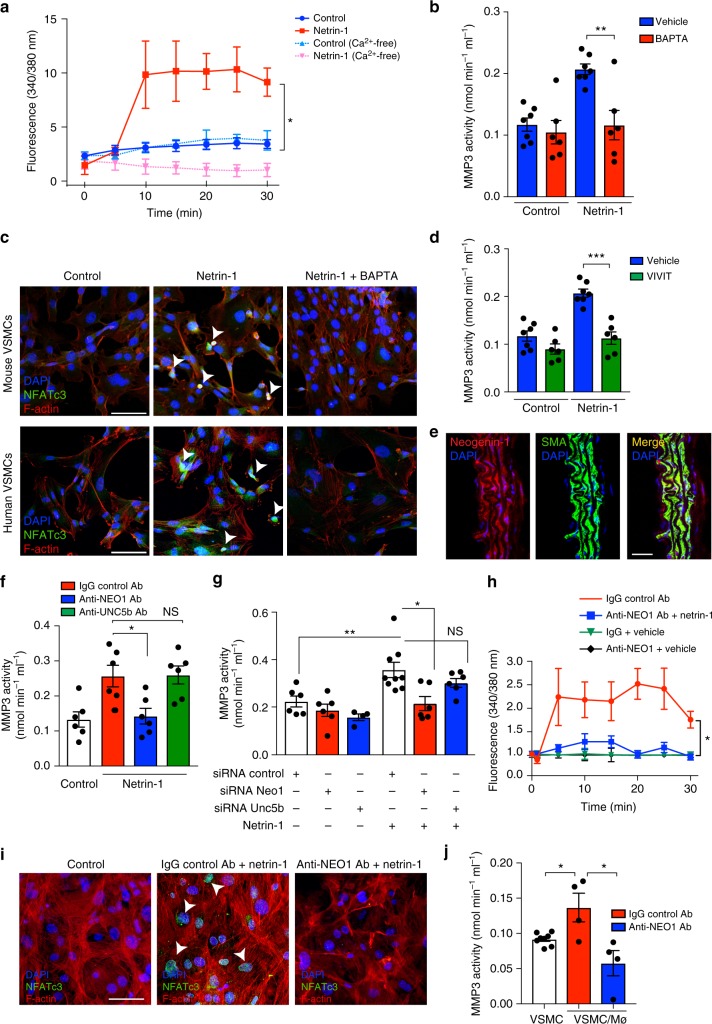
Netrin-1 mobilizes Ca^2+^ and drives MMP3 activity in vascular smooth muscle cells via its receptor neogenin-1. **a** Calcium integration curves in VSMCs stimulation as indicated. Data are expressed as 340/380 nm excitation ratio. *n* = 4. *P < 0.05. **b** MMP3 activity in murine VSMCs stimulated with netrin-1 in the presence or absence of calcium chelator BAPTA (10 µM). *n* = 6–7 per group. **P < 0.01. **c** NFATc3 (green) and actin (F-actin, red) immunofluorescence staining in mouse VSMCs (top panel) or human VSMCs (lower panel) stimulated with netrin-1 (2.5 µg/ml) with or without BAPTA. White arrowheads indicate nuclear location, *n* = 4. Scale bars 20 µm. **d** MMP3 activity in mouse VSMCs stimulated with netrin-1 in the presence or absence of NFATc3 inhibitor, VIVIT (1 µM). *n* = 6–7 per group. ***P < 0.001. **e** Immunofluorescence staining of neogenin-1 (NEO-1, red) and alpha-smooth muscle actin (SMA, green) in aortic sections of *ApoE*^*−/−*^ mice. Colocalization is shown in merge. Nuclei stained with DAPI (blue). Scale bar 50 µm. **f** MMP3 activity in VSMCs stimulated with recombinant netrin-1 in the presence of control IgG, anti-NEO1, or anti-UNC5B neutralizing antibody (10 µg/ml). *n* = 6 per group. *P < 0.05, NS not significant. **g** MMP3 activity in VSMCs transfected with siRNA control, Neo1 or Unc5b and stimulated with recombinant netrin-1. *n* = 4–9 per group. *P < 0.05, **P < 0.01, NS = not significant. **h** Calcium integration curves in VSMCs stimulated as indicated. Data are expressed as 340/380 nm excitation ratio. *P < 0.05. **i** NFATc3 and F-actin immunofluorescence staining of VSMCs stimulated with netrin-1, in the presence of control IgG or anti-NEO1 blocking antibody. White arrowheads indicate nuclear translocations. Scale bar 20 µm. **j** MMP3 enzymatic activity in VSMCs co-cultured with WTMø in the presence of control IgG or anti-NEO1 antibody. *n* = 4–8 per group. *P < 0.05. Two-way ANOVA was used to assess statistical significance in **a** and **h**. Unpaired, two-tailed *t*-test was used for statistical analysis in **b**, **d**, **f**, **g**, and **j**. Error bars represent s.e.m

We further delved into the molecular mechanisms regulating netrin-1-dependent effects on MMP3 in VSMCs. The decoding of the sequenced RNA-Seq library through pathway enrichment predicted a crosstalk between netrin-1 and calcium signaling in conjunction with MMP3 expression (Supplementary Figure 2b). Notably, calcium (Ca^2+^) is a critical co-factor necessary for the activation of the catalytic subunit of MMP3^35^. Because excessive secretion of extracellular matrix degrading enzyme can be due to intracellular accumulation of Ca^2+^, we questioned whether netrin-1 could impact the availability of Ca^2+^. We found that netrin-1 potently and rapidly induced an intracellular Ca^2+^ wave in VSMCs as tracked by excitation emitted by the Ca^2+^-sensitive FURA-2-fluorescent dye (Fig. 6a). Similar experiments in the presence of Ca^2+^-free media abrogated the boost of intracellular Ca^2+^ suggesting that exogenous netrin-1 could regulate the mobilization of extracellular pools of Ca^2+^. Accordingly, stimulation of VSMCs with netrin-1 in the presence of the Ca^2+^ chelator, 1,2-bis(o-aminophenoxy) ethane -N,N,N′,N′-tetraacetic acid (BAPTA) blunted the induction of MMP3 activity (Fig. 6b). Immunofluorescence of murine and human VSMCs following netrin-1 stimulation revealed the nuclear translocation of NFATc3, a transcription factor previously described to regulate MMP3 expression in astrocytes^35^. The staining pattern was reversed in the presence of BAPTA suggesting that netrin-1-induced NFATc3 nuclear translocation is dependent on Ca^2+^ (Fig. 6c). Importantly, netrin-1-induced MMP3 catalytic activity was totally abrogated in the presence of NFATc3 inhibitor, MAGPHPVIVITGPHEE (VIVIT) peptide (Fig. 6d). Altogether these findings suggested the central role for netrin-1 in driving MMP3 activity by synchronizing Ca^2+^ signaling in VSMCs.

### Netrin-1 regulates MMP3 via its receptor neogenin-1 in VSMCs

We have previously demonstrated that neogenin-1 is the primary receptor for netrin-1 expressed by VSMCs^21^. Here, we tested whether netrin-1 could regulate MMP3 expression via  neogenin-1. Immunoflurescence staining revealed a colocalized expression of neogenin-1 and VSMCs in murine aortic sections (Fig. 6e). MMP3 activity was increased in VSMCs stimulated with recombinant netrin-1 but not in cells pretreated with anti-neogenin-1 blocking antibody. The exposure of VSMCs to anti-Unc5b, another reported receptor of  netrin-1, did not abrogate netrin-1-induced MMP3 activity compared to cells exposed to IgG control (Fig. 6f). We validated these results by a second method of neogenin-1 invalidation by siRNA-mediated approach. Stimulation of VSMCs with recombinant netrin-1 induced the activity of MMP3 in VSMCs exposed to non-targeting scrambled siRNA (siRNA control) while the silencing of neogenin-1, not *Unc5b*, abrogated the activity of MMP3 in the presence of recombinant netrin-1 (Fig. 6g). These data suggested that the selective activation of neogenin-1 by netrin-1 triggers signaling cascades required to activate MMP3. Notably, netrin-1-induced intracellular Ca^2+^ wave in VSMCs was blunted in VSMCs exposed to anti-neogenin-1 blocking antibody (Fig. 6h). The nuclear translocation of the transcription factor NFATc3 induced by netrin-1 was refrained in cells exposed to neogenin-1 blocking antibodies (Fig. 6i). To demonstrate that macrophage-derived netrin-1 could regulate MMP3 via neogenin-1 in VSMCs, we performed co-culture assays between macrophages and VSMCs in transwells. MMP3 activity was increased in VSMCs co-cultured with activated macrophages, while it was significantly reduced in VSMCs exposed to anti-neogenin-1 blocking antibody (Fig. 6j). Taken together, we have identified that netrin-1 secreted by macrophages can act as a central catalyzer of MMP3 activity via neogenin-1 activation in VSMCs.

## Discussion

Our results have uncovered dynamic mechanisms that bridge sustained inflammation and focal ECM erosion in the AAA sac and identified netrin-1 as an instrumental signal in perpetuating this destructive response as illustrated in the supplementary schematic (Supplementary Figure 4).

Netrin-1 regulated intracellular calcium flux in VSMCs. This finding has not yet been described and is important as calcium is a key cofactor necessary to maintain cellular homeostasis^36,37^ in the vascular wall. This is in accordance with studies that initially uncovered the role of netrin-1 in the neuronal system^38^. Notably, netrin-1 could induce calcium by mobilizing intracellular stores, a mechanism required for axonal branching^39^. Despite that some reports have evoked aberrant calcium signaling in AAA, the direct role of calcium in the focal activation of MMP3 was undescribed so far. Our data provide evidence that netrin-1 can direct the extracellular calcium influx into VSMCs as calcium measurements performed in the presence of calcium-free media completely blunted netrin-1-induced signal. Interestingly, aberrant calcium regulation which leads to calcium depots has been described to be causative in vascular diseases such as atherosclerosis^40^ and Marfan syndrome^41^. In the latter, extensive elastin fragmentation was shown to manifest in areas enriched in microcalcification^42^. Future studies are required to uncover the exact molecular signaling pathways through which netrin-1 can exert such effects on the import of calcium in cells. Notably, it is likely that netrin-1 could synchronize intracellular pools of calcium that might orchestrate VSMCs function in a broad spectrum. Interestingly, emerging reports have described the plasticity of VSMCs within injured aortic tissues^43^. VSMCs can deviate from their original contractile phenotype and adopt a macrophage-like secretory hybrid role. This is tightly dependent on the microenvironment. VSMCs can also inherit enhanced contractile potential such as those isolated from mice with Marfan syndrome^44^. Our results indicate that the exposure of VSMCs to netrin-1, robustly drive calcium signaling critical for the proteolytic activity of MMP3.

Consistent with other studies, increased MMP3 expression has been described in AAA^45^. Elevated levels of MMP3 can be attributable in part to the influx of activated inflammatory cells including monocytes/macrophages within the aneurysmal tissue^46^. Depletion of these phagocytic cells dampened enzymatic activities in the diseased portion of the aorta^18,47^. Our results demonstrate that netrin-1 induced MMP3 activity specifically in VSMCs but not in endothelial cells nor in fibroblasts. This can be explained by the uneven distribution of the receptor of netrin-1, neogenin-1 in vascular cells. As such, our data indicate that the activation of neogenin-1 expressed by VSMCs leads to intracellular Ca^2+^/NFATc3 mobilization responsible to regulate MMP3 catalytic activity. Furthermore, besides MMP3 uncontrolled catalytic activity in AAA, independent studies have uncovered that the gene encoding MMP3 bears a functional polymorphism characterized by adenosine number (5A/6A) in the promoter region^48,49^ and represent independent risk factors for AAA^50^, further emphasizing the involvement of this enzyme in the pathogenesis of AAA. However, the direct role of MMP3 in AAA was not addressed so far in the gold-standard Ang II-infused murine model. Amidst the investigation of the role of MMP3 in atherosclerosis, Lijnen et al showed that the deletion of MMP3 in *ApoE*^*−/−*^ mice exposed to high cholesterol diet reduced features of aortic damage, but did not protect against lesion development^51^. These findings are in agreement with our results demonstrating that the deletion of MMP3 in AAA-prone mice reduced arterial damage associated with AAA development.

The current dogma generally supports the paradigm that inflammatory cells might contribute to AAA by promoting the exaggerated activity of MMPs causative of significant vascular injury precipitating to aortic expansion and rupture^18^. However, the role of these enzymes was studied secondary to the manipulation of immune subsets shadowing the contribution of other stromal cells within the AAA sac. Notably, recent concepts depicting the role of MMPs in directing inflammatory signals have emerged^52–54^ suggesting that vascular cells producing MMPs might also impact local inflammation. Our data surmise that macrophages synergize with VSMCs to shape ECM breakdown unrestrictedly most likely by obstructing mechanisms responsible for the repair of ECM. Noteworthy, pharmacological suppression of MMPs such as doxycycline therapies resulted not only in reduced aortic MMP3 expression but also dampened the inflammatory response in a clinical cohort^55^ raising the question of whether the contribution of inflammation to AAA is secondary to vascular injury or whether it is the origin of sequences that converge to damage in AAA. Interestingly, in experimental models of AAA, doxycycline administration failed to refrain the progression of established AAA^56^ suggesting that additional factors contribute to the initiation of the disease. Our data provide insights of how macrophages that invade AAA leave an imprint of their presence within the arterial scar which is causative to the timely and sustained activation of MMP3. This is strongly supported by our results demonstrating that the absence of MMP3 in macrophages is insufficient to protect against AAA in contrast to the impact of netrin-1 deficiency in macrophages. However, our in vitro data supported that netrin-1 could direct MMP3 activity in macrophages. While the reasons for these discrepant findings are unclear, they may be explained because in *Mmp3*^*−/−*^→WT bone-marrow chimeras, VSMCs might compensate for the loss of MMP3 in macrophages henceforth masking the protective effect of macrophage-derived MMP3 in *Mmp3*^*−/−*^→WT bone-marrow chimeras. Furthermore, MMP3 can act as an upstream activator of other MMP isoforms including MMP9^57,58^. Interestingly, studies have highlighted the deleterious role of macrophage-derived MMP9 in AAA^59,60^. Therefore, MMP3 produced by VSMCs could contribute to enhance macrophage-induced ECM degradation even though the latter are refractory to MMP3 expression. We can therefore speculate that macrophages recruited to the site of aortic trauma might serve as an initial damage trigger which eventually perpetuates into chronic MMP3 catalytic signals following VSMCs activation by netrin-1.

Taken together, our study provides important mechanistic evidence of how macrophage-derived netrin-1 can impact focal MMP3 activation. We anticipate that interventions to disrupt such crosstalks might be the cornerstone of therapeutic strategies for future drug development in the setting of AAA.

## Methods

### Mice

C57BL/6J (WT), LysMcre and *ApoE*^*−/−*^ mice were purchased from Jackson Laboratories. *Ntn1*^+/−^ mice were bred with *ApoE*^*−/−*^ to generate *Ntn1*^+/+^*ApoE*^*−/−*^ and *Ntn1*^−/−^*ApoE*^*−/−*^ donors. Fetal liver cells (2 × 10^6^) from donor mice were injected intravenously into 6−week-old recipient *ApoE*^*−/−*^ mice that were lethally irradiated (two exposures of 600 cGy) followed by antibiotic treatment. Reconstituted mice were allowed to recover for 6 weeks before experimentation. LysMcre^+/−^ mice were bred with *Ntn1*^flox/flox^ mice (provided by Dr. Holger Eltzschig) to generate LysMcre^+/−^*Ntn1*^flox/flox^ mice to delete *Ntn1* in LysMcre positive lineage. *Ntn1*^flox/flox^ littermates were used as experimental controls. MMP3-deficient (*Mmp3*^*−/−*^) mice on C57BL/6J background were purchased from Taconic Biosciences. Lethally irradiated C57BL/6 J mice were reconstituted with either C57BL/6J or *Mmp3*^*−/−*^ bone marrow cells following lethal irradiation to generate WT→WT and *Mmp3*^*−/−*^→WT chimeras. For stromal deletion of MMP3 studies, *Mmp3*^*−/−*^ mice were used as recipient and transplanted with bone marrow cells from C57BL/6J to generate WT→*Mmp3*^*−/−*^ chimeras. All mice were maintained in a pathogen-free facility. C57BL/6J mice were fed a standard chow or Western diet (WD; 21% [wt/wt] fat, 0.3% cholesterol; Research Diets). When applicable, mice were distributed randomly in each group and sample size has not been predetermined by statistical method. Mice dying before sacrifice with no signs of aortic pathology were excluded from the analyses. Experimental procedures were approved and completed in accordance with guidelines set forth in the US Department of Agriculture Animal Welfare Act, the Public Health Service Policy for the Humane Care and Use of Laboratory Animals and the New York University School of Medicine’s Institutional Animal Care and Use Committee.

### Human samples

The studies were approved and conducted in accordance with policies of the New York University Langone Medical Center Institutional Review Board. Informed consent was obtained for each subject. Samples of unruptured human aneurysmal (>50 mm) aortic wall were collected during open aortic repair procedures. After orientation of the tissue following macroscopic analysis, samples were formalin-fixed and paraffin-embedded prior to sectioning. For laser capture microdissection, frozen serial sections were stained for CD68 and CD206 to identify CD68^+^CD206^−^ and CD68^+^CD206^+^ macrophage regions, respectively. CD68^+^CD206^−^ and CD68^+^CD206^+^ enriched unstained 18 µm sections were isolated from the appropriate areas on CapSure Macro LCM Caps using an ArcturusXT Microdissection Instrument (MDS Analytical Technologies) and processed immediately for RNA extraction with the PicoPure RNA Extraction Kit (KIT0204, MDS Analytical Technologies). RNA integrity was checked on an Agilent 2100 Bioanalyzer (Agilent Technologies) with RNA Pico Chips (Agilent Technologies) and amplified 2 rounds through the ExpressArt Trinucleotide mRNA amplification Nano Kit (7299-A15, AMS-Biotechnology) before retro-transcription^27^.

### AAA induction in mice

Alzet osmotic pumps (model 2004; 0000298, Durect Corporation) were loaded with PBS or angiotensin II (H-1705, Bachem) and implanted subcutaneously for a delivery rate of 1 μg/kg/min for 28 days as previously described^15^. Blood pressure was monitored weekly by tail cuff using the CODA machine (Kent Scientific Corporation), and aneurysm progression was assessed by ultrasound Doppler imaging. At sacrifice, macroscopic evaluation of the aorta was used to grade the severity of AAA^26^. Enlarged aorta without thrombus and dilated aorta that frequently contained thrombus was scored as stages I and II, respectively. Stage III categories included bulbous aortas with visible thrombus and stage IV was defined as multiple aneurysmal containing thrombus. Prior to aneurysm induction, 6–8-week-old mice were injected with 10^11^ genomic copies of either PCSK9 or control AAV based on the study of Daugherty et al.^30^ and fed a Western diet as described above until sacrifice.

### Ultrasound imaging

A Vevo 2100 ultrasound imaging platform (FUJIFILM VisualSonics) was utilized throughout the study. After sedation, mice were placed on a heating pad and kept on sedation throughout the procedure. Body temperature and cardiac pace were monitored. Mice were placed on an electric-heated blanket to maintain normothermia and continuously delivered a gas inhalation of two percent isoflurane. Heart rate was monitored throughout image acquisition and kept at a consistent range (400–500 beats per minute) as previously described^61–63^, and provided reproducible results for all experimental groups. Mice abdomen were shaved and Aquasonic 100 ultrasound transmission gel (NC9861677, Parker Laboratories) was added to increase probe contact. All images were acquired using the settings provided by the manufacturer. The probe was first applied on short axis to locate the aorta, and the pulsatile property of the vessel was used to distinguish from the vena cava (PW mode). The aorta was then centered and the probe switched to the long axis. Color mode Doppler was then activated to help localize the two renal arteries, displaying a red blood flow toward the probe on the screen. The probe was moved until the aorta was strictly parallel to the device in order to acquire measurements of the aortic diameter. Three measurements were performed by a blinded investigator on the long axis (M mode): above the upper renal artery, on maximal aortic dilation, and below the lower renal artery. Data for the maximal transverse aortic diameter is shown for individual animals.

### RNA sequencing

RNA was extracted from aortas using the RNEasy fibrous tissue kit as per manufacturer’s instructions (74704, Qiagen). RNA quality was verified using a Bioanalyzer (G2939BA, Agilent Technologies). The samples were run on a HiSeq (Illumina) as single-end reads, 50 nucleotides in length. FASTQ files were aligned to the MM9 Mus musculus reference genome using Tophat (version 2.0.9) with two mismatches allowed. The resulting read counts were extracted using the Featurecounts program from binary alignment map (BAM) files. Differential gene expression analysis was conducted using the DESEQ2 package from the Bioconductor repository using the open source R statistical programming environment. All downstream data manipulation, plotting, and statistical filtering were also performed in the same environment using custom scripts. *P* values attained from differential gene expression analysis were adjusted for multiple testing by controlling for false discovery using the Benjamini-Hochberg method and genes with adjusted *P* values < 0.05 were flagged as differentially expressed then used for Gene Set Enrichment Analysis (GSEA). GSEA was performed using the DAVID Bioinformatics Resource (version 6.8) to elucidate relevant biological significance.

### Single-cell RNA-sequencing

Aortas were collected and digested for 1 h at 37 °C in an enzymatic mix (10 mg/ml Collagenase type II (C6885, Sigma Aldrich) and 1 mg/ml Elastase (LS002292, Worthington Biochemistry). Digested aortas were mechanically disrupted and immediately processed for single-cell RNA-sequencing. The cellular suspensions were loaded on a 10x Genomics Chromium instrument to generate single cell gel beads in emulsion (GEMs). Libraries were prepared using the following kits: Chromium Single Cell 3′ Library & Gel Bead Kit v2, PN-120237; Single Cell 3′ Chip Kit v2 PN-120236 and i7 Multiplex Kit PN-120262, 10x Genomics) as described^64^. Sequencing was performed on an Illumina HiSeq 4000 as 2 x 150 paired-end reads, one full lane per sample, for approximately >90% sequencing saturation. For alignment, barcode assignment and unique molecular identifiers (UMI) counting, the Cell Ranger Single Cell Software Suite, version 1.3 was used to perform sample de-multiplexing, barcode and UMI processing, and single-cell 3′ gene counting (https://support.10xgenomics.com/single-cell-gene). Data analysis was performed using the Loupe Cell Browser software (10x Genomics) on Cloupe files displaying *t*SNE projections of cell transcriptome. Cell types were identified by their expression levels of gene provided that at least two copies of the transcript were found in the cell (cutoff: log_2_ fold-increased copies = 1 vs 1 copy only per cell) (Supplementary Table 1). Loupe Cell Browser was then used to compare the transcriptome of each identified cluster corrected for false discovery rates, as applied in RNA sequencing. The expression of transcripts within macrophage clusters identified as *Ntn-1* positive or negative subclusters are provided in supplementary Table 2.

### Real-time quantitative PCR

Samples were homogenized in TRIzol reagent (15596026, Ambion, Life Technologies) and mRNA was extracted using a Direct-zol RNA MiniPrep Kit (R2052, Zymo Research). Retro-transcription in cDNA was performed with the iScript cDNA Synthesis Kit (1708890, Bio-Rad). Quantitative real-time PCR was then carried out with KAPA SYBR FAST qPCR Kits (KK4602, KAPA Biosystems) on a QuantStudio 3 Real-Time PCR System (Applied Biosystems) in triplicates. Results were analyzed and fold change over housekeeping gene were calculated by the comparative cycle method (2^−ΔΔCt^).

Primer sequences used:

Mouse *28S*: F TGGGAATGCAGCCCAAG, R CCTTACGGTACTTGTTGACTATCG

Mouse *Ntn1*: F CAGCCTGATCCTTGCTCGG, R GCGGGTTATTGAGGTCGGTG

Mouse *Adgre1*: F CCCCAGTGTCCTTACAGAGTG, R GTGCCCAGAGTGGATGTCT

Mouse *Ccl2*: F TTAAAAACCTTGGATCGGAACCAA, R GCATTAGCTTCAGATTTACGGGT

Mouse *Il6*: F CCAAGAGGTGAGTGCTTCCC, R CTGTTGTTCAGACTCTCTCCCT

Mouse *Mmp3*: F GTCCCTCTATGGAACTCCCAC, R AGTCCTGAGAGATTTGCGCC

Human *28S*: F TGGGAATGCAGCCCAAG, R CCTTACGGTACTTGTTGACTATCG

Human *MRC1*: F CGAGGAAGAGGTTCGGTTCAC C, R GCAATCCCGGTTCTCATGGC

Human *IL1B:* F AGCTCGCCAGTGAAATGATGG, R CAGGTCCTGGAAGGAGCACTTC

Human *NTN1*: F ACAACCCGCACAACCTGAC, R GGGACAGTGTGAGCGTGAC

### Cell culture

Mice femurs and tibias were collected and the bone marrow was flushed and differentiated into primary BMDMs. Cells were cultured for 7 days in DMEM (MT10013CV, Fisher Scientific) supplemented with 10% Fetal Bovine Serum (FBS; 10082147, Life Technologies),1% penicillin–streptomycin (PS; 30-002-CI, Corning) and 20% L-929-conditioned media. After differentiation, BMDMs were stimulated as indicated in results. Mouse primary vascular smooth muscle cells (VSMCs) were isolated from the descending section of freshly dissected aortas. After dissection and removal of the peri-adventitial fat, aortic samples were digested for 10 min at 37 °C in enzymatic mix (10 mg/ml type II collagenase, C6885, Sigma Aldrich and 1 mg/ml elastase, LS002292, Worthington Biochemistry). The adventitia was gently removed under a dissecting microscope and the remaining tissue was digested for 50 additional minutes in the enzymatic mix. Digested aortas were then mechanically disrupted and passed through a 70 µm cell strainer (340607, BD Biosciences). Cells were then cultured in DMEM-10%FBS-1%PS and used for experiments as previously described^65^. VSMCs lineage was confirmed by the presence of immunoreactivity for α-actin by flow cytometry (Alexa Fluor 405 anti-alpha smooth muscle actin; ab210128, Abcam). Human primary vascular smooth muscle cells (CRL-1999^TM^, ATCC) and mouse embryonic fibroblasts (CRL-2991^TM^, ATCC), were purchased from the American Type Culture Collection and cultured according to manufacturer’s instruction. For subculturing, mycoplasma contamination was tested by a commercially available kit (M7006, Invitrogen) as per the manufacturer’s instructions.

Pharmacological inhibitors, VIVIT (MAGPHPVIVITGPHEE peptide, #3905, Tocris Biosciences) or BAPTA (B1205, Invitrogen), were added 30 min prior netrin-1 stimulation. In some assays, neutralizing antibodies, goat anti-neogenin-1 antibody (AF1079-SP, R&D Systems), goat anti-Unc5b antibody (AF1006-SP, R&D Systems) or goat IgG control (AB-108-C, R&D Systems) were added 30 min prior to stimulations. To ensure reproducibility, three technical replicates were performed each time on at least two biologically different cell lines.

For co-culture experiments, BMDMs from WT or *Ntn1*^flox/flox^LysMcre^+/−    ^Ntn1 mice (10^5^ cells) were cultivated on top chamber of transwell inserts, in 200 µl DMEM 2% FBS. Mouse WT VSMC were seeded in the lower chamber of Corning™ Transwell™ Multiple Well Plate with Permeable Polycarbonate Membrane Inserts (07-200-147, ThermoFisher), 10^5^ cells/well in 500 µl DMEM 10% FBS, and allowed to adhere overnight. BMDM/VSMC were then co-cultured for 24 h and VSMC were lysed in 50 µl RIPA buffer (without protease inhibitor) and assayed for Mmp3 activity.

To knock down neogenin-1 and Unc5b, VSMCs were transfected with siRNA targeting neogenin-1 (Neo 1 siRNA SASI_Mm02_00291095, Sigma Aldrich) or Unc5b (E-050737-00-0005, Dharmacon, SMARTpool) using siRNA reagent system (sc-45064, Santacruz Biotechnology) as per manufacturers’ instructions. A non-targeting siRNA-scrambled sequence was as control (SC-37007, Santacruz Biotechnology).

### MMP3 activity assay

MMP3 activity in cells and homogenized aortic tissue was assessed using the MMP3 Activity Fluorimetric Assay Kit (K783–100, Biovision), following the manufacturer’s instruction. Data were expressed as the amount of enzyme generating 1 µmol of fluorescent substrate per minute per milliliter of sample (nmol/min/ml).

### Flow cytometry

Aortic cell suspensions were prepared as described above. Digested aortas were then mechanically disrupted and passed through a 70 µm cell strainer (340607, BD Bioscience), centrifuged 10 min at 1000 × *g* at room temperature and fixed in 300 µl PBS-2%PFA before further staining. A volume of 50 µl of aortic cell suspension was then stained for 20 min with an antibody mix consisting of VioGreen^TM^ anti-CD45 (130-102-412, Miltenyi biotechnology) PE-Vio770 anti-F4/80 (130-102-193, Miltenyi biotechnology) and APC anti-CD11b (130-091-241, Miltenyi biotechnology), 1:50 dilution each, resuspended in 500 µl of PBS-1%PFA, ran on a BD LSRII flow cytometer (Becton Dickinson) recorded using the FACSDiva software (Becton Dickinson) and acquired data were analyzed using the FlowJo software (FlowJo, LLC). Doublets were excluded on a FSC-A/FSC-H dot plots. Leukocyte population, which served as a stopping gate (5000 events recorded in the stopping gate), was identified as CD45 positive cells on a FSC-A/CD45 dot plots. Macrophage population within leukocytes was identified as a CD11b^+^F4/80^+^ populations on a CD11b/F4/80 plots (on average, 500 (for the PBS-infused mice) to 2500 (for the Ang II-infused mice) macrophages were recorded).

### Immunohistochemical staining

Mice aortas were included in Optimal Cutting Temperature Compound (OCT; 4585, Fisher Scientific) and 7-µm-thick sections were cut and fixed with 10% formalin buffer. Human paraffin-embedded aortas sections were deparaffinized and rehydrated by successive washes of xylene, xylene/ethanol (vol/vol), 100% ethanol, 95% ethanol, 70% ethanol and water. Antigen retrieval was performed by boiling in 10 mM Tris, 1 mM EDTA, 0.05% Tween20, pH9 retrieval buffer. Sections were then blocked before overnight incubation of primary antibodies: chicken anti-Mouse/human netrin-1 (ab39370, Abcam), rat anti-Mouse CD68 (MCA1957, Bio-Rad), mouse anti-Human CD68 (MCA5709, Bio-Rad), rabbit anti-mouse/human MMP3 (ab53015, Abcam), goat anti -mouse neogenin-1 (AF1079, R&D Systems) or anti-mouse alpha-smooth muscle actin (ab7817, Abcam) (1:500 dilution each). Secondary fluorescent antibodies (Alexa Fluor 488 goat anti-mouse, A11001, Alexa Fluor 568 goat anti-mouse, A11004, Alexa Fluor 488 goat anti-rabbit, A11008, Alexa Fluor 568 goat anti-rabbit, A11011, Alexa Fluor 568 goat anti-rat, A11077, Alexa Fluor 568 donkey anti-goat, A-11057, Alexa Fluor 488 goat anti-chicken, A11039, 1:400 dilution each, Invitrogen) were then applied for detection. VSMCs were seeded directly on Lab-Tek chamber slides (154534, Thermo Fisher Scientific), 5 × 10^5^ cells/well in complete DMEM. Following netrin-1 (1109-N1, R&D Systems) stimulation (2.5 µg/ml), cells were fixed in ice-cold 2% buffered formalin, blocked and permeabilized before incubation with primary mouse anti-NFATc3 antibody (1:200 dilution; SC-8405, Santa Cruz Biotechnology). Secondary fluorescent Alexa-Fluor 488 goat anti-mouse antibody (1:500 dilution; A11001, Invitrogen) was then applied for detection. 4′,6-diamidino-2-phénylindole (DAPI, 1: 50,000; D1306, Invitrogen) was used for cell nucleus staining. Alexa-Fluor 568 phalloidin (1:40 dilution; A12380, Invitrogen) was used to stain actin cytoskeleton. Images were acquired using a Zeiss LSM 700 confocal microscope (Carl Zeiss) through the Zeiss Efficient Navigation (ZEN) software (Carl Zeiss). Identical acquisition parameters were set to capture control and test samples. Quantifications were performed on sections from three different animals without any prior setting modification. Levels of brightness and contrast were adjusted following identical modifications within each experiment. Scale bars are indicated on respective magnified images for each experiment.

For histochemical assays, 7 µm mice aortic sections included in OCT were stained for elastin figments using the Verhoeff Van Gieson Elastin stain set (25089-1, Astral Diagnostics) according to the manufacturer’s instructions. Seven micrometer human aortic sections were deparaffinized as aforementioned and hematoxylin-eosin (H&E) staining was performed by incubating the slides 1.5 min in eosin 515 LT (3801619, Leica Biosystems) and 2 min in hematoxylin 560 MX (3801575, Leica Biosystems).

### Western blotting

Samples were homogenized in RIPA buffer (98065; Cell Signaling Technologies). Forty micrograms of denatured proteins were loaded onto a 10% Mini-PROTEAN TGX gel (#456-8034, Bio-Rad) for SDS-PAGE. Proteins were transferred on PVDF membranes (#BR20160719, Bio-Rad) using a Trans-Blot Turbo Transfer System (Bio-Rad). Membranes were blocked and probed for netrin-1 (1:500 dilution; ab126729, Abcam) and GAPDH (1:1000 dilution; ab8245, Abcam). Secondary biotinylated antibodies goat anti-rabbit (1:5000 dilution; A0545, Sigma Aldrich) or goat anti-mouse (1:5000 dilution; A9917, Sigma Aldrich) were applied before revelation with Clarity Western ECL (#170-5060, Bio-Rad) and imaged on a ChemiDoc Imaging System (Bio-Rad). Uncropped blot images are shown in Supplementary Figure 6. Mean netrin-1 band intensity was measured with ImageJ and normalized over GAPDH mean band intensity.

### Calcium imaging

Mice primary VSMCs were seeded in 96-Well Clear Bottom Black Polystyrene Microplates (3603; Corning) at a density of 2 × 10^5^ cells/well. The cells were allowed to adhere for 24 h in complete DMEM medium at 37 °C and 5% CO_2_. Cells were then loaded for 30 min with Calcium tracker Fura-2 (1 μM; F1221, Thermo Fisher Scientific). Cells were washed and incubated for an additional 15 min in PBS. A volume of 100 µl of DMEM or DMEM without Calcium (21068028; Thermo Fisher Scientific) was then added to each well. Baseline fluorescence was acquired on a FlexStation 3® Multi-Mode Microplate Reader (Molecular Devices). Light emission was acquired at 550 nm following excitation at 340 (Calcium-bound Fura-2 max excitation peak) and 380 nm (Calcium-free Fura-2 max excitation peak). Recombinant mouse netrin-1 (1109-N1, R&D Systems) was then directly loaded to each well (final concentration 2.5 µg/ml), and changes in fluorescence max excitation peak were monitored for 30 min. Data were expressed as the ratio of values captured at 340 nm and 380 nm wavelengths (340/380 ratio).

### Statistical analysis

Categorical data are presented as percentages. Continuous variables are shown as the means±s.e.m. Two-tailed, unpaired Student’s *t*-test was used to compare two groups of continuous data. Comparisons among more than two independent groups were analyzed by one-way or two-way analysis of variance (ANOVA), followed by Tukey or Dunnett multiple comparison tests when appropriate. Two-sided Fisher’s exact test has been used to compare categorical variables. Linear regression was computed for two-variable correlation. Goodness of fit and 95% interval bands are as indicated. Statistical significance of Kaplan-Meier survival curves was assessed with a Mantel-Cox test. Analyses were performed with Prism 7 software (GraphPad software Inc., CA, USA). *P* values < 0.05 were considered significant.

## Electronic supplementary material

Supplementary File

## Data Availability

The data and computer codes that support our findings are available from the corresponding author upon reasonable request. All RNA sequencing datasets are deposited in Gene Expression Omnibus (GEO)-accession number GSE118237 (GSM3323357 for the CLOUPE file used to generate Fig. 3a–d and f and the Supplementary Tables 1 and 2) and GSE118217 (GSM3321219 to GSM3321226 for each individual sample used to generate Fig. 4a and b, and Supplementary Figure 2a and b). A reporting summary for this Article is available as a Supplementary Information file.
