# Supplementary material for: Macrophage-derived netrin-1 promotes abdominal aortic aneurysm formation by activating MMP3 in vascular smooth muscle cells
Source: Nat Commun. 2018 Nov 27;9:5022. doi: 10.1038/s41467-018-07495-1 (PMC6258757; doi:10.1038/s41467-018-07495-1)
Supplement: Supplementary file 1 — Supplementary File [file 41467_2018_7495_MOESM1_ESM.pdf]

Supplementary Information

"Netrin-1 promotes Abdominal Aortic Aneurysms by sustaining the activation of MMP3 in the vascular wall"

Hadi et al.

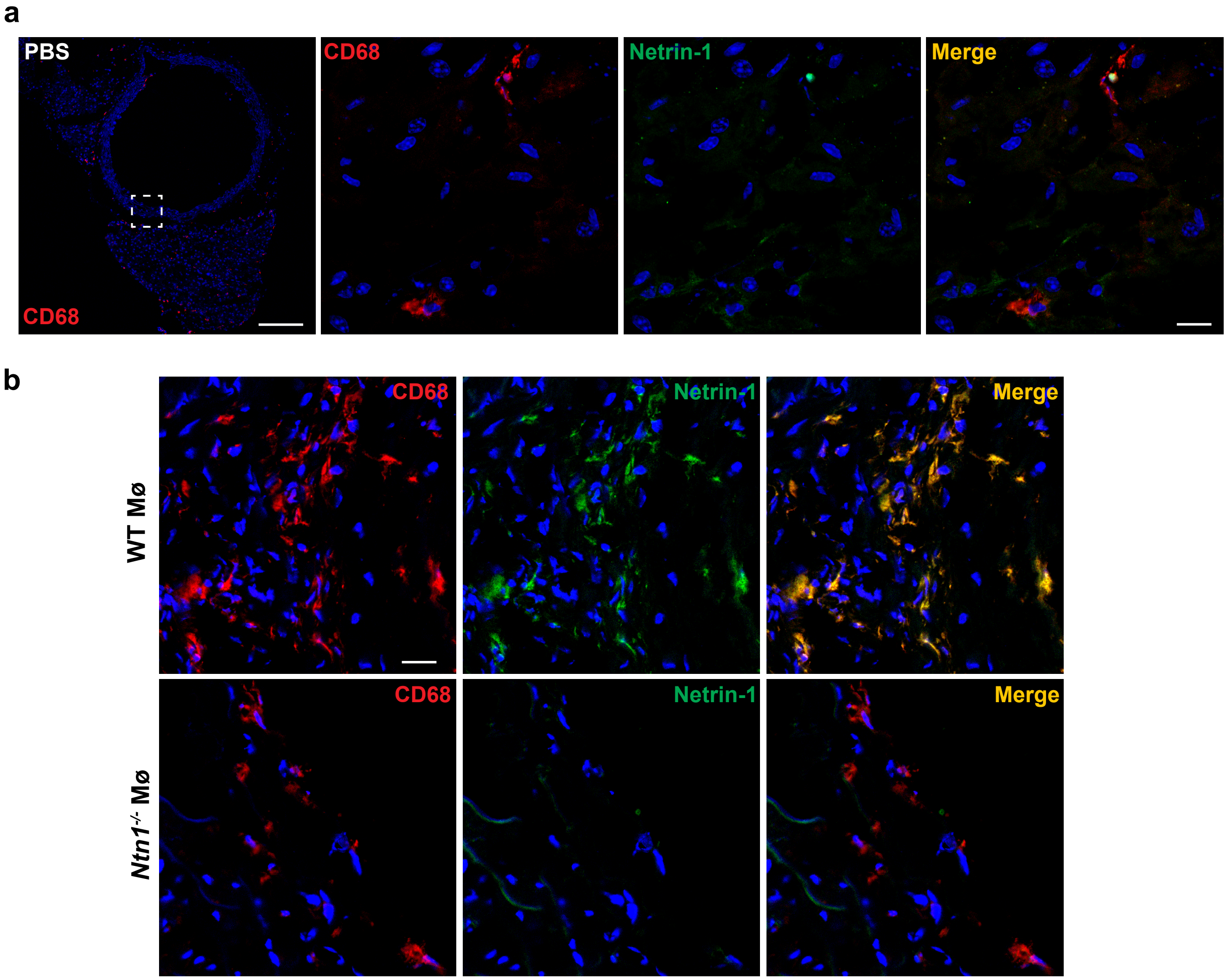

Supplementary figure 1: Netrin-1 is absent in healthy aortas and deleted in aortic macrophages of *Ntn1*$^{flox/flox}$*LysM-Cre*$^{+/-}$ mice

(a) Representative immunofluorescence staining of CD68 and netrin-1 in aortic sections of mice exposed to PBS. n= 3. Scale bar left panel 200 µm, right panels 20 µm. Nuclei stained with DAPI (blue). **(b)** Representative immunofluorescence staining of CD68 (red) and netrin-1 (green) and their colocalization (yellow) in aortic sections of *Ntn1*$^{flox/flox}$*LysMcre*$^{-/-}$ (WT Mø) and *Ntn1*$^{flox/flox}$*LysMcre*$^{+/-}$ (*Ntn1*$^{-/-}$ Mø) mice treated with Ang II for 28 days. n= 3 mice per group. Scale bar 20 µm.

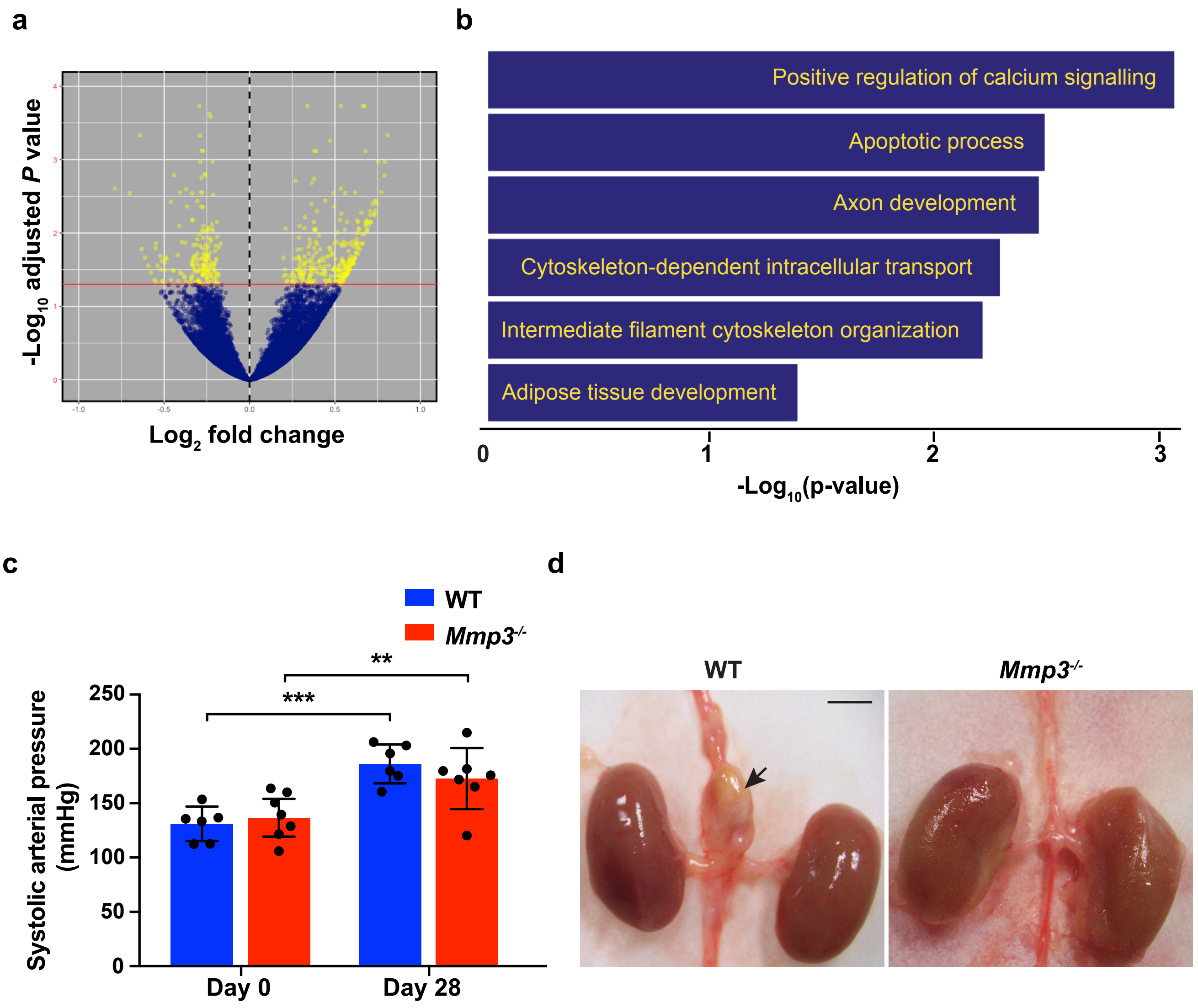

Supplementary figure 2: Netrin-1 regulates MMP3 activity in AAA

Volcano plot representation showing $-\log_{10}$ adjusted P values and $\log_2$ fold change of transcriptome profiling **(a)** and pathway analysis using the DAVID software displaying relevant pathways in WT -> *ApoE*$^{-/-}$ compared to *Ntn1*$^{-/-}$ -> *ApoE*$^{-/-}$ aorta **(b)**. n= 4 in each group. Systolic arterial pressure of WT and *Mmp3*$^{-/-}$ mice exposed to Ang II for 0 or 28 days **(c)** and representative photomicrograph of aortas at harvest **(d)**. n= 6-7 mice per group. Scale bar 2 mm. Statistical significance assessed with unpaired, two-tailed *t*-test. $^{**}P < 0.01$, $^{***}P < 0.001$. Error bars represent s.e.m.

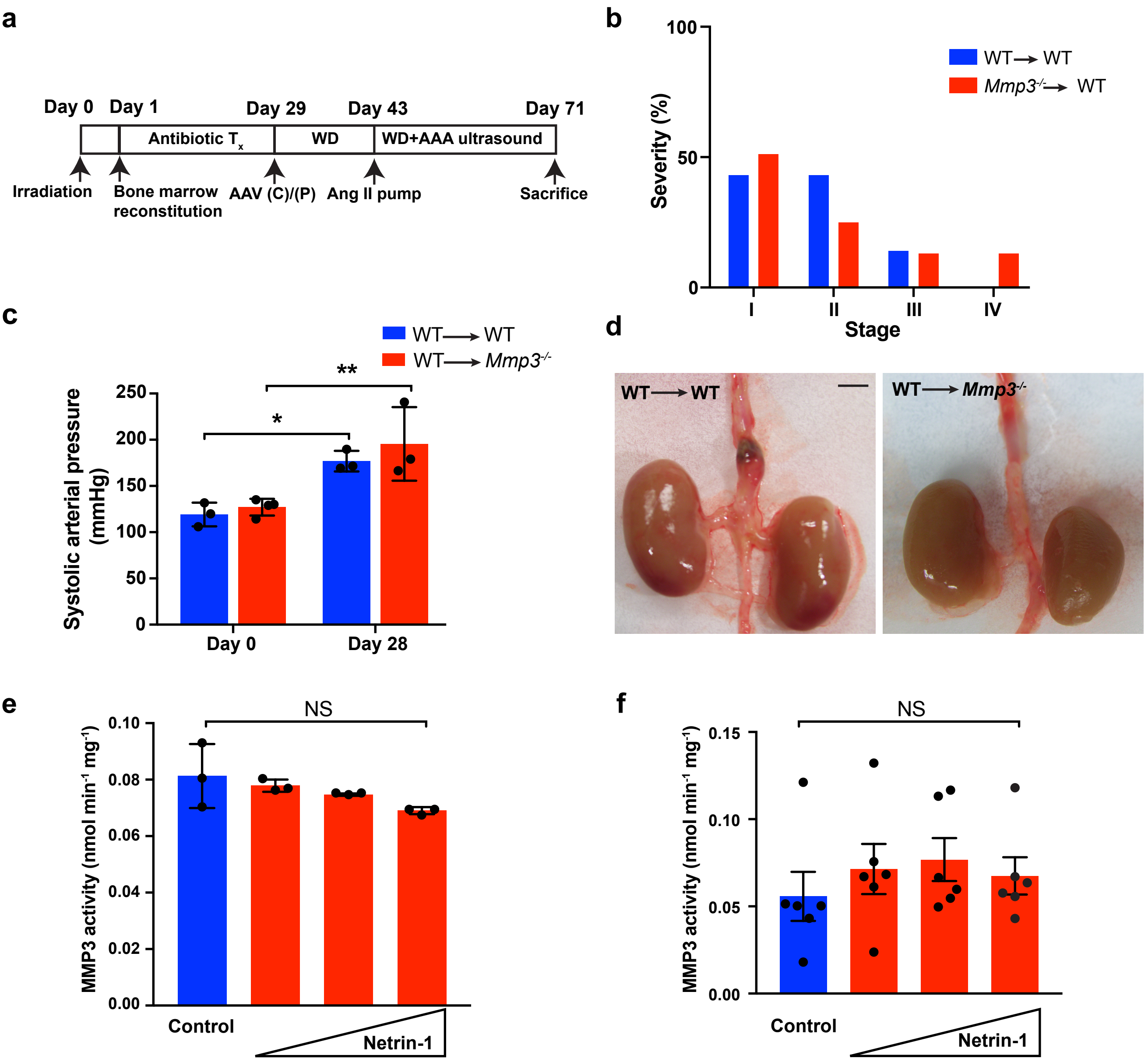

Supplementary figure 3: MMP3 deficiency in vascular smooth muscle cells, but not in macrophages, protects against AAA development

(a) Schematic timeline representation of irradiation and bone marrow reconstitution, followed by antibiotics treatment (Tx), PCSK9 (P) or control (C) adeno-associated virus vector (AAV) injection and Ang II infusion. WD, western diet. **(b)** Severity stratification of aneurysms of WT recipient mice irradiated and reconstituted with WT (WT -> WT) or *Mmp3*$^{-/-}$ (*Mmp3*$^{-/-}$ -> WT) bone marrow, exposed to Ang II for 28 days. Arterial pressure measurement of WT -> WT or WT -> *Mmp3*$^{-/-}$ challenged to Ang II at day 0 and day 28 **(c)** and representative photomicrograph of aortas at harvest **(d)**. n= 6 mice per group. Scale bar 2 mm. *P < 0.05, **P < 0.01. MMP3 activity in coronary artery endothelial cells **(e)** and in mouse embryonic fibroblasts **(f)** stimulated with 0.625, 1.25 or 2.5 µg/mL of recombinant netrin-1. n=3-6 per group. NS, not significant. Unpaired, two-tailed t-test was used for statistical analysis in (c). One-way ANOVA was used in (e) and (f). Error bars represent s.e.m.

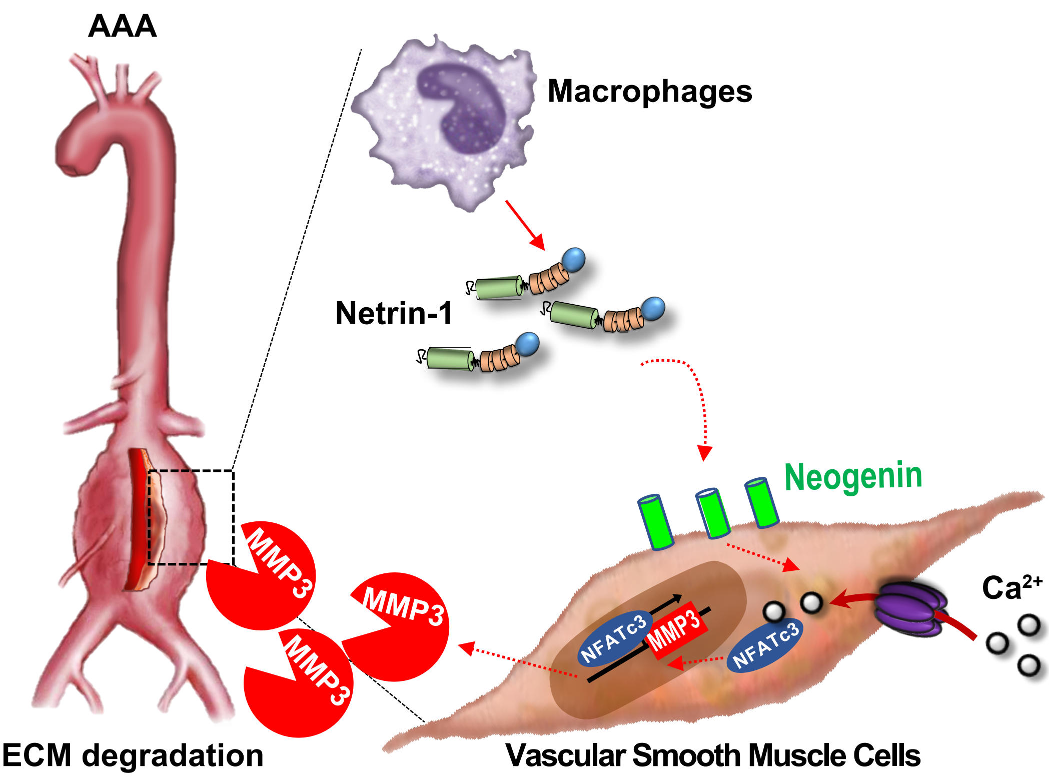

Supplementary figure 4: Schematic representation of our main findings

Macrophages that accumulate within the aortic wall secrete netrin-1. Netrin-1 induces intracellular flux of Ca^{2+} required for the activation of NFATc3 nuclear translocation *via* its receptor neogenin-1 in vascular smooth muscle cells. Consequently, MMP3 is activated and degrades its substrates within the extra-cellular matrix (ECM) thereby fueling chronic ECM injury and leading to AAA progression.

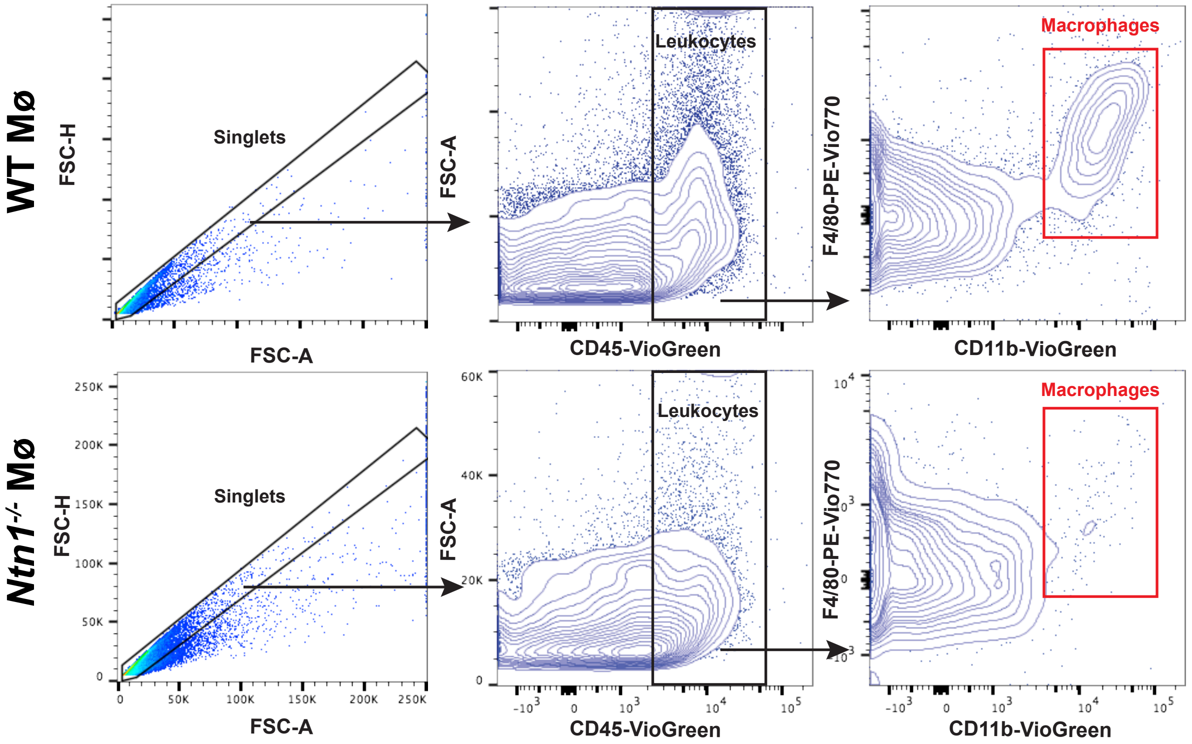

Supplementary figure 5: Flow cytometry gating strategy

Representative dot plots and gating of WTMø (upper panels) or *Ntn1*$^{-/-}$Mø (lower panels) for the macrophage quantification used in figure 3m. Singlets were gated on a FSC-A/FSC-H dot plots (black boxes, left panel). Leukocyte population (black boxes, middle panels) was identified as CD45 positive cells on a FSC-A/CD45 dot plots. Macrophage population (red boxes, right panels) within leukocytes was identified as a CD11b+F4/80+ populations on a CD11b/F4/80 plot. (n= 6; WT and *Ntn1*$^{-/-}$ Mø/AAV P).

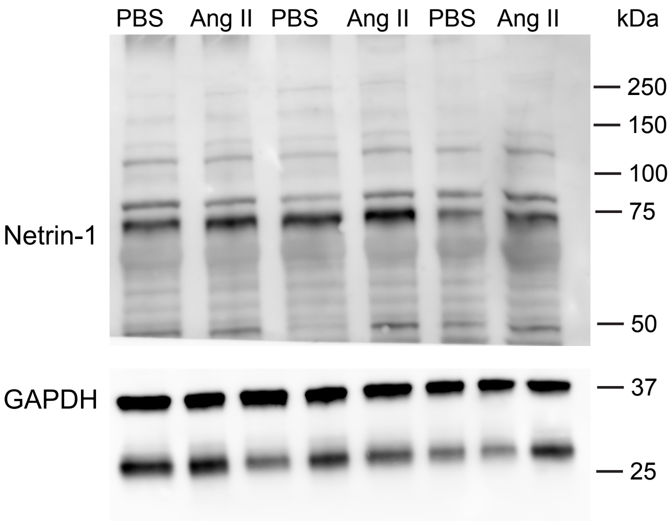

Supplementary Figure 6: Original images of Western Blots used in the study. Blots show indicated proteins and molecular weight marker from figure 1b.

EnsemblID	GeneName	Macrophages Log2 Fold Change	Macrophages P-Value	B Cells Log2 Fold Change	B Cells P-Value	T Cells Log2 Fold Change	T Cells P-Value	Dendritic cells Log2 Fold Change	Dendritic cells P-Value	Neutrophils Log2 Fold Change	Neutrophils P-Value
ENSMUSG00000029373	Pf4	5.83	1.19E-14	-1.92	1.00E+00	-1.92	1.00E+00	-1.58	1.00E+00	0.59	1.00E+00
ENSMUSG00000018774	Cd68	5.63	5.93E-19	0.7	1.00E+00	-0.31	1.00E+00	5.8	5.01E-02	2.21	1.00E+00
ENSMUSG00000036887	C1qa	5.5	4.68E-20	-2.96	1.00E+00	-1.96	1.00E+00	4.35	3.72E-01	-0.44	1.00E+00
ENSMUSG00000036896	C1qc	5.45	1.03E-19	-2.59	1.00E+00	-1.59	1.00E+00	4.62	2.65E-01	-0.08	1.00E+00
ENSMUSG00000039109	F13a1	5.39	1.15E-11	-0.53	1.00E+00	-0.54	1.00E+00	-0.2	1.00E+00	1.98	1.00E+00
ENSMUSG00000069516	Lyz2	5.29	2.34E-19	-2.54	1.00E+00	-3.13	1.00E+00	5.05	1.34E-01	-1.62	1.00E+00
ENSMUSG00000036905	C1qb	5.25	2.34E-19	-2.95	1.00E+00	-2.96	1.00E+00	4.94	1.67E-01	-0.44	1.00E+00
ENSMUSG00000061100	Retnla	5.09	5.44E-02	-0.35	1.00E+00	0.65	1.00E+00	-0.01	1.00E+00	2.16	1.00E+00
ENSMUSG00000018927	Ccl6	4.97	1.13E-11	-1.72	1.00E+00	-0.72	1.00E+00	2.09	1.00E+00	0.79	1.00E+00
ENSMUSG00000044811	AF251705	4.85	1.69E-10	1.73	1.00E+00	1.73	1.00E+00	6.25	5.01E-02	4.24	1.00E+00
ENSMUSG00000030579	Tyrobp	4.84	4.06E-19	-1.21	1.00E+00	-2.22	1.00E+00	4.85	1.34E-01	0.3	1.00E+00
ENSMUSG00000058715	Fcer1g	4.79	1.25E-18	-2.19	1.00E+00	-2.2	1.00E+00	5.17	8.13E-02	0.32	1.00E+00
ENSMUSG00000049723	Mmp12	4.7	6.73E-02	0.94	1.00E+00	0.94	1.00E+00	6.4	3.33E-01	3.46	1.00E+00
ENSMUSG00000089672	Lilr4b	4.55	9.84E-07	1.83	1.00E+00	1.82	1.00E+00	6.48	6.52E-02	4.34	1.00E+00
ENSMUSG00000019122	Ccl9	4.52	4.98E-11	-1.16	1.00E+00	-0.16	1.00E+00	3.12	8.76E-01	1.35	1.00E+00
ENSMUSG00000046805	Mpeg1	4.5	4.94E-10	1.29	1.00E+00	0.28	1.00E+00	6.39	3.55E-02	2.79	1.00E+00
ENSMUSG00000059089	Fcgr4	4.37	3.67E-03	2.68	1.00E+00	2.68	1.00E+00	6.49	2.30E-01	5.19	1.00E+00
ENSMUSG00000009185	Ccl8	4.32	9.75E-04	-1.81	1.00E+00	-1.81	1.00E+00	0.11	1.00E+00	0.7	1.00E+00
ENSMUSG00000069792	Wfdc17	4.29	3.56E-09	-0.28	1.00E+00	-0.28	1.00E+00	4.97	2.56E-01	0.64	1.00E+00
ENSMUSG00000038642	Ctss	4.09	1.45E-10	2.72	1.00E+00	1.31	1.00E+00	6.46	5.91E-03	0.11	1.00E+00
ENSMUSG00000028238	Atp6v0d2	3.61	4.92E-02	2.24	1.00E+00	2.24	1.00E+00	7.14	1.07E-01	4.75	1.00E+00
ENSMUSG00000052336	Cx3cr1	3.57	7.98E-03	1.6	1.00E+00	1.6	1.00E+00	7.34	4.67E-02	4.11	1.00E+00
ENSMUSG00000027398	Il1b	3.32	3.27E-01	3.07	1.00E+00	3.68	9.18E-01	6.24	3.87E-01	4.56	1.00E+00
ENSMUSG00000028581	Laptm5	3.32	3.59E-11	3.49	5.51E-01	3.29	6.17E-01	5.37	2.74E-02	1.36	1.00E+00
ENSMUSG00000089942	Pira2	3.2	2.12E-02	4.55	1.00E+00	3.49	1.00E+00	7.82	5.91E-03	6	1.00E+00
ENSMUSG00000040564	Apoc1	3.19	1.04E-03	-1.62	1.00E+00	-1.62	1.00E+00	-1.28	1.00E+00	0.89	1.00E+00
ENSMUSG00000030560	Ctsc	2.98	6.12E-09	1.46	1.00E+00	1.04	1.00E+00	2.39	1.00E+00	0.96	1.00E+00
ENSMUSG00000028459	Cd72	2.82	6.10E-03	4.29	6.31E-01	1.98	1.00E+00	6.26	5.16E-02	2.9	1.00E+00
ENSMUSG00000026358	Rgs1	2.75	4.00E-02	2.03	1.00E+00	3.66	1.00E+00	6.36	8.21E-02	4.54	1.00E+00
ENSMUSG00000025473	Adam8	2.73	5.50E-02	2.46	1.00E+00	2.46	1.00E+00	7.32	2.18E-02	4.97	1.00E+00
ENSMUSG00000032554	Trf	2.71	7.46E-07	1.19	1.00E+00	-2.14	1.00E+00	1.37	1.00E+00	1.37	9.92E-01
ENSMUSG00000050335	Lgals3	2.68	5.49E-04	-3.15	1.00E+00	-3.15	1.00E+00	5.08	1.35E-01	-0.64	1.00E+00
ENSMUSG00000021880	Rnase6	2.36	8.86E-02	6.45	1.29E-02	2.37	1.00E+00	4.35	8.33E-01	4.88	1.00E+00
ENSMUSG00000022582	Ly6g	2.32	1.00E+00	7.07	1.00E+00	7.07	1.00E+00	7.41	1.00E+00	11.58	9.92E-01
ENSMUSG00000051506	Wdfy4	2.32	2.29E-01	4.07	1.00E+00	2.43	1.00E+00	6.19	1.99E-01	4.94	1.00E+00
ENSMUSG00000109028	RP23-449M8.6	1.99	7.58E-01	5.49	1.00E+00	5.49	1.00E+00	5.82	1.00E+00	9.26	9.92E-01
ENSMUSG00000010142	Tnfrsf13b	1.99	1.14E-01	6.07	1.00E-02	1.96	1.00E+00	3.93	9.40E-01	4.48	1.00E+00
ENSMUSG00000022512	Cldn1	1.81	4.95E-01	3.61	1.00E+00	3.61	1.00E+00	3.95	1.00E+00	7.19	9.92E-01
ENSMUSG00000035385	Ccl2	1.77	4.34E-01	0.9	1.00E+00	0.9	1.00E+00	1.24	1.00E+00	3.41	1.00E+00
ENSMUSG00000022496	Tnfrsf17	1.73	5.99E-01	9.66	8.73E-03	3.32	1.00E+00	3.65	1.00E+00	5.83	1.00E+00
ENSMUSG00000094777	Hist1h2ap	1.71	4.01E-01	3.92	1.00E+00	1.26	1.00E+00	6.52	7.99E-02	3.78	1.00E+00
ENSMUSG00000038179	Slamf7	1.64	4.45E-01	7.47	1.32E-03	3.75	1.00E+00	6.17	1.27E-01	4.63	1.00E+00
ENSMUSG00000073293	Nudt10	1.61	6.05E-01	4.07	1.00E+00	4.07	1.00E+00	4.41	1.00E+00	7.68	9.92E-01
ENSMUSG00000097280	AI849053	1.32	1.00E+00	6.07	1.00E+00	6.07	1.00E+00	6.41	1.00E+00	10	9.92E-01
ENSMUSG00000030787	Lyve1	1.14	5.04E-01	-1.4	1.00E+00	-1.4	1.00E+00	-1.06	1.00E+00	1.12	1.00E+00
ENSMUSG00000029816	Gpnmb	1.12	3.29E-01	-2.76	1.00E+00	-1.76	1.00E+00	6.25	7.79E-04	-0.25	1.00E+00
ENSMUSG00000030165	Klrd1	1.02	6.23E-01	2.22	1.00E+00	6.52	5.62E-02	4.63	6.95E-01	4.73	1.00E+00
ENSMUSG00000087166	L1td1	0.99	1.00E+00	5.75	1.00E+00	5.75	1.00E+00	6.09	1.00E+00	9.58	9.92E-01
ENSMUSG00000015854	Cd5l	0.92	6.43E-01	2.2	1.00E+00	1.19	1.00E+00	8.97	6.53E-03	3.7	1.00E+00
ENSMUSG00000030724	Cd19	0.91	7.15E-01	7.94	4.05E-05	2.64	1.00E+00	2.98	1.00E+00	5.16	1.00E+00
ENSMUSG00000086644	Gm13470	0.87	7.34E-01	3.65	1.00E+00	2.61	1.00E+00	2.95	1.00E+00	6.16	9.92E-01
ENSMUSG00000034379	Wdr5b	0.87	6.63E-01	2.24	1.00E+00	3.26	1.00E+00	2.58	1.00E+00	5.78	9.92E-01
ENSMUSG00000101493	2810405F17Rik	0.86	6.90E-01	3.43	1.00E+00	3.43	1.00E+00	2.74	1.00E+00	5.94	9.92E-01
ENSMUSG00000027420	Bfsp1	0.79	6.33E-01	1.75	1.00E+00	1.75	1.00E+00	2.09	1.00E+00	5.88	9.92E-01
ENSMUSG00000003882	Il7r	0.79	6.58E-01	1.24	1.00E+00	6.5	2.25E-02	5.02	3.96E-01	3.75	1.00E+00
ENSMUSG00000037849	Gm4955	0.73	7.29E-01	4.37	9.46E-01	6.75	1.26E-02	2.63	1.00E+00	4.8	1.00E+00
ENSMUSG00000041000	Trim62	0.65	8.39E-01	2.65	1.00E+00	2.64	1.00E+00	2.98	1.00E+00	6.19	9.92E-01
ENSMUSG00000029212	Gabrb1	0.51	1.00E+00	5.27	1.00E+00	5.26	1.00E+00	5.6	1.00E+00	9	9.92E-01
ENSMUSG00000040936	Ulk4	0.51	1.00E+00	3.12	1.00E+00	3.12	1.00E+00	3.46	1.00E+00	6.68	9.92E-01
ENSMUSG00000066677	Pydc3	0.47	8.70E-01	2.75	1.00E+00	6.62	2.33E-02	3.09	1.00E+00	5.26	1.00E+00
ENSMUSG00000026185	Igfbp5	0.45	6.65E-01	-5.59	1.00E+00	-3.59	1.00E+00	-4.25	1.00E+00	0.63	9.92E-01
ENSMUSG00000078552	Dcdc2b	0.44	1.00E+00	3.49	1.00E+00	3.49	1.00E+00	3.82	1.00E+00	7.06	9.92E-01
ENSMUSG00000032232	Cgnl1	0.41	7.02E-01	-0.44	1.00E+00	-1.44	1.00E+00	-0.1	1.00E+00	1.07	1.00E+00
ENSMUSG00000030789	Itgax	0.38	8.39E-01	3.43	1.00E+00	3.43	1.00E+00	10.15	7.79E-04	5.94	1.00E+00
ENSMUSG00000010797	Wnt2	0.37	7.58E-01	-0.21	1.00E+00	-0.21	1.00E+00	0.12	1.00E+00	2.3	1.00E+00
ENSMUSG00000028040	Efna4	0.35	9.78E-01	2.37	1.00E+00	2.37	1.00E+00	4.35	7.19E-01	5.91	9.92E-01
ENSMUSG00000047281	Sfn	0.35	9.99E-01	6.14	8.03E-03	4.01	1.00E+00	2.71	1.00E+00	4.88	1.00E+00
ENSMUSG00000022951	Rcan1	0.34	7.72E-01	-0.69	1.00E+00	-0.69	1.00E+00	-0.35	1.00E+00	1.82	1.00E+00
ENSMUSG00000026833	Olfm1	0.33	7.51E-01	-1.38	1.00E+00	-1.39	1.00E+00	1.77	1.00E+00	1.13	1.00E+00
ENSMUSG00000026344	Lypd1	0.32	9.58E-01	2.12	1.00E+00	2.12	1.00E+00	2.46	1.00E+00	6.26	9.92E-01
ENSMUSG00000038421	Fcrla	0.27	9.33E-01	5.97	4.82E-02	1.88	1.00E+00	3.24	1.00E+00	4.39	1.00E+00
ENSMUSG00000034205	Loxl2	0.21	8.48E-01	-2.21	1.00E+00	-2.22	1.00E+00	-0.29	1.00E+00	0.3	1.00E+00
ENSMUSG00000029201	Ugdh	0.21	8.46E-01	-1.44	1.00E+00	-0.44	1.00E+00	-0.1	1.00E+00	0.07	1.00E+00
ENSMUSG00000041482	Piezo2	0.17	9.13E-01	-1.29	1.00E+00	-1.29	1.00E+00	0.05	1.00E+00	1.22	1.00E+00
ENSMUSG00000051951	Xkr4	0.15	1.00E+00	3.83	1.00E+00	3.82	1.00E+00	4.16	1.00E+00	7.41	9.92E-01
ENSMUSG00000034634	Ly6d	0.15	9.13E-01	6.04	8.52E-03	1.31	1.00E+00	1.65	1.00E+00	2.82	1.00E+00
ENSMUSG00000030468	Siglecg	0.13	1.00E+00	6.05	2.53E-02	1.75	1.00E+00	2.09	1.00E+00	4.26	1.00E+00
ENSMUSG00000034390	Cmip	0.09	9.81E-01	-0.51	1.00E+00	0.08	1.00E+00	0.83	1.00E+00	1	1.00E+00
ENSMUSG00000028480	Glipr2	0.08	1.00E+00	1.12	1.00E+00	2.12	1.00E+00	0.86	1.00E+00	2.03	1.00E+00
ENSMUSG00000022838	Eaf2	0.07	9.91E-01	8.35	2.57E-04	2.71	1.00E+00	3.05	1.00E+00	5.23	1.00E+00
ENSMUSG00000027030	Stk39	0.05	1.00E+00	-0.88	1.00E+00	-0.89	1.00E+00	-0.55	1.00E+00	1.63	1.00E+00
ENSMUSG00000021391	Cenpp	-0.08	1.00E+00	3.61	1.00E+00	3.61	1.00E+00	6.56	7.53E-02	7.19	9.92E-01
ENSMUSG00000038587	Akap12	-0.11	1.00E+00	-1.06	1.00E+00	-1.06	1.00E+00	-1.72	1.00E+00	1.45	9.92E-01
ENSMUSG00000017002	Slpi	-0.12	7.17E-01	9.6	2.37E-02	-2.06	1.00E+00	-1.72	1.00E+00	0.45	1.00E+00
ENSMUSG00000027611	Procr	-0.15	1.00E+00	-1.37	1.00E+00	-1.37	1.00E+00	-0.03	1.00E+00	1.14	1.00E+00
ENSMUSG00000020902	Ntn1	-0.17	1.00E+00	-2.53	1.00E+00	-2.53	1.00E+00	-2.19	1.00E+00	-0.01	1.00E+00
ENSMUSG00000030830	Itgal	-0.39	1.00E+00	5.49	2.22E-01	6.75	5.90E-03	4.09	1.00E+00	5.23	1.00E+00
ENSMUSG00000037548	H2-DMb2	-0.4	1.00E+00	6.3	3.58E-03	0.8	1.00E+00	2.15	1.00E+00	3.32	1.00E+00
ENSMUSG00000048776	Pthlh	-0.4	1.00E+00	-1.2	1.00E+00	-0.2	1.00E+00	0.14	1.00E+00	2.31	9.92E-01
ENSMUSG00000032053	Pou2af1	-0.42	1.00E+00	8.55	5.61E-05	2.68	1.00E+00	3.02	1.00E+00	5.19	1.00E+00
ENSMUSG00000024673	Ms4a1	-0.58	1.00E+00	6.27	8.03E-03	1.4	1.00E+00	0.73	1.00E+00	2.91	1.00E+00
ENSMUSG00000022033	Pbk	-0.62	1.00E+00	2.49	1.00E+00	2.49	1.00E+00	6.26	2.20E-01	5	1.00E+00
ENSMUSG00000003379	Cd79a	-0.66	1.00E+00	6.3	1.37E-03	-0.99	1.00E+00	-0.65	1.00E+00	1.53	1.00E+00
ENSMUSG00000087131	Gm16152	-0.68	9.28E-01	4.07	1.00E+00	4.07	1.00E+00	3.37	1.00E+00	6.58	9.92E-01
ENSMUSG00000052180	Serpinb6c	-1.14	6.83E-01	2.58	1.00E+00	2.58	1.00E+00	2.92	1.00E+00	6.13	9.92E-01
ENSMUSG00000040592	Cd79b	-1.21	7.79E-01	6.32	1.32E-03	1.28	1.00E+00	0.02	1.00E+00	2.2	1.00E+00

ENSMUSG00000022584	Ly6c2	-1.42	9.91E-01	8.73	1.32E-03	4.39	7.12E-01	0.17	1.00E+00	2.34	1.00E+00
ENSMUSG00000009092	Derl3	-1.44	1.00E+00	9.4	2.79E-03	3.32	1.00E+00	2.63	1.00E+00	4.8	1.00E+00
ENSMUSG00000032093	Cd3e	-1.44	8.62E-01	2.29	1.00E+00	9.27	9.30E-10	2.63	1.00E+00	4.8	1.00E+00
ENSMUSG00000035042	Ccl5	-1.48	9.42E-01	0.77	1.00E+00	7.24	1.02E-01	0.11	1.00E+00	2.28	1.00E+00
ENSMUSG00000000409	Lck	-1.61	6.83E-01	2.55	1.00E+00	6.68	1.00E-03	1.87	1.00E+00	4.05	1.00E+00
ENSMUSG00000027863	Cd2	-1.67	7.08E-01	4.13	7.98E-01	6.37	9.30E-03	1.81	1.00E+00	3.98	1.00E+00
ENSMUSG00000030742	Lat	-1.71	7.25E-01	2.03	1.00E+00	7.04	1.27E-03	2.37	1.00E+00	4.54	1.00E+00
ENSMUSG00000053977	Cd8a	-1.93	9.78E-01	2.83	1.00E+00	8.75	8.95E-05	3.16	1.00E+00	5.34	1.00E+00
ENSMUSG00000054641	Mmrn1	-1.98	8.53E-01	-0.25	1.00E+00	-1.26	1.00E+00	-0.92	1.00E+00	1.26	1.00E+00
ENSMUSG00000054892	Txk	-2.04	7.14E-01	2.72	1.00E+00	6.58	2.44E-02	3.05	1.00E+00	5.23	1.00E+00
ENSMUSG00000094686	Ccl21a	-2.05	9.60E-01	-4.39	1.00E+00	-4.39	1.00E+00	-3.05	1.00E+00	-1.87	1.00E+00
ENSMUSG00000053044	Cd8b1	-2.31	8.78E-01	1.43	1.00E+00	8.5	3.43E-05	1.77	1.00E+00	3.94	1.00E+00
ENSMUSG00000026989	Dapl1	-2.33	1.00E+00	2.43	1.00E+00	7.9	5.52E-03	2.77	1.00E+00	4.94	1.00E+00
ENSMUSG00000032094	Cd3d	-2.35	6.21E-01	0.81	1.00E+00	7.6	2.22E-05	1.14	1.00E+00	3.32	1.00E+00
ENSMUSG00000027985	Lef1	-2.41	6.65E-01	2.35	1.00E+00	6.69	1.34E-02	2.68	1.00E+00	4.86	1.00E+00
ENSMUSG00000031933	Izumo1r	-2.73	1.00E+00	3.05	1.00E+00	6.73	8.11E-02	2.37	1.00E+00	4.54	1.00E+00
ENSMUSG00000009145	Dqx1	-2.89	1.00E+00	1.86	1.00E+00	1.86	1.00E+00	2.2	1.00E+00	4.38	1.00E+00
ENSMUSG00000004612	Nkg7	-3.44	1.00E+00	2.33	1.00E+00	6.74	6.78E-02	1.65	1.00E+00	3.83	1.00E+00
ENSMUSG00000024353	Mzb1	-3.45	1.00E+00	10.2	3.52E-06	0.3	1.00E+00	-0.37	1.00E+00	1.81	1.00E+00
ENSMUSG00000002033	Cd3g	-3.8	4.31E-01	0.95	1.00E+00	7.44	5.60E-05	1.29	1.00E+00	3.47	1.00E+00
ENSMUSG00000056290	Ms4a4b	-4.26	3.52E-01	0.5	1.00E+00	7.07	5.74E-04	0.83	1.00E+00	3.01	1.00E+00
ENSMUSG00000067149	Jchain	-4.95	9.42E-01	12.99	2.83E-03	-1.79	1.00E+00	-2.03	9.49E-01	-0.86	1.00E+00

Supplementary table 1: **Immune cell clustering and analysis of Ntn1 expression**

List of transcript levels in immune cell clusters. Data are expressed as $\log_2$ fold change and *P* values for gene in each cluster are shown.

	EnsemblID	GeneName	Netrin Low Log2 Fold Change	Netrin Low P-Value	Netrin High Log2 Fold Change	Netrin High P-Value
Increased in Netrin high macrophages	ENSMUSG00000026691	Fmo3	-3.8	1.70E-03	3.83	8.75E-04
	ENSMUSG00000020902	Ntn1	-3.7	3.35E-10	3.72	2.46E-10
	ENSMUSG00000019851	Perp	-3.65	2.41E-02	3.68	1.87E-02
	ENSMUSG00000028989	Angptl7	-3.3	1.17E-01	3.33	9.84E-02
	ENSMUSG00000023064	Sncg	-3.11	5.78E-02	3.13	4.65E-02
	ENSMUSG00000042379	Esm1	-3.09	2.25E-01	3.11	1.99E-01
	ENSMUSG00000026208	Des	-3.07	1.22E-01	3.09	1.03E-01
	ENSMUSG00000024990	Rbp4	-2.97	2.23E-02	2.99	1.59E-02
	ENSMUSG00000043613	Mmp3	-2.95	1.31E-02	2.96	8.54E-03
	ENSMUSG00000028128	F3	-2.89	1.08E-03	2.91	5.54E-04
	ENSMUSG00000032744	Heyl	-2.77	9.66E-03	2.8	5.54E-03
	ENSMUSG00000097324	Mir143hg	-2.68	9.17E-03	2.7	5.20E-03
	ENSMUSG00000026768	Itga8	-2.62	1.39E-02	2.65	9.20E-03
	ENSMUSG00000039542	Ncam1	-2.61	7.06E-03	2.63	2.88E-03
	ENSMUSG00000020019	Ntn4	-2.47	1.45E-02	2.5	1.03E-02
	ENSMUSG00000053684	BC048403	-3.86	3.16E-01	2.47	8.81E-01
	ENSMUSG00000037362	Nov	-2.35	1.39E-02	2.38	9.20E-03
	ENSMUSG00000026255	Efhd1	-2.34	2.85E-02	2.36	2.06E-02
	ENSMUSG00000001349	Cnn1	-2.32	4.27E-02	2.35	3.52E-02
	ENSMUSG00000014846	Tppp3	-2.33	2.23E-02	2.35	1.59E-02
	ENSMUSG00000006219	Fblim1	-2.34	8.48E-03	2.35	5.20E-03
	ENSMUSG00000027811	4930579G24Rik	-2.45	9.31E-01	1.3	1.00E+00
	ENSMUSG00000062944	9130023H24Rik	-2.45	9.12E-01	1.3	1.00E+00
	ENSMUSG00000105699	Gm43703	-2.86	1.00E+00	0.88	1.00E+00
	ENSMUSG00000071083	Gm10311	-2.86	1.00E+00	0.88	1.00E+00
	ENSMUSG00000035860	Cdhr3	-2.86	1.00E+00	0.88	1.00E+00
	ENSMUSG00000103686	Gm37073	-2.86	1.00E+00	0.88	1.00E+00
	ENSMUSG00000074634	Gm7120	-2.86	1.00E+00	0.88	1.00E+00
	ENSMUSG00000097429	Gm26520	-1.86	1.00E+00	0.3	1.00E+00
	ENSMUSG00000044566	Cage1	-1.86	1.00E+00	0.3	1.00E+00
	ENSMUSG00000093514	Gm28727	-1.86	1.00E+00	0.3	1.00E+00
	ENSMUSG00000026065	Slc9a4	-1.86	1.00E+00	0.3	1.00E+00
	ENSMUSG00000026068	Il18rap	-1.86	1.00E+00	0.3	1.00E+00
	ENSMUSG00000097558	Gm26902	-1.86	1.00E+00	0.3	1.00E+00
Decreased in Netrin high macrophages	ENSMUSG00000101903	Gm29291	-1.28	1.00E+00	-0.12	1.00E+00
	ENSMUSG00000052142	Rasal3	-1.28	1.00E+00	-0.12	1.00E+00
	ENSMUSG00000054702	Ap1s3	-1.28	1.00E+00	-0.12	1.00E+00
	ENSMUSG00000030789	Itgax	-1.86	1.00E+00	-0.44	1.00E+00
	ENSMUSG00000022494	Shisa9	-1.86	1.00E+00	-0.44	1.00E+00
	ENSMUSG00000030117	Gdf3	-4.67	5.25E-01	-0.92	1.00E+00
	ENSMUSG00000027513	Pck1	4.72	1.88E-01	-4.69	1.88E-01
	ENSMUSG00000037071	Scd1	4.7	5.26E-01	-4.71	5.27E-01
	ENSMUSG00000032725	Folr2	4.78	2.33E-02	-4.75	1.83E-02
	ENSMUSG00000031250	Tnmd	4.84	1.00E+00	-4.82	1.00E+00
	ENSMUSG00000002944	Cd36	5.01	1.08E-03	-4.99	7.85E-04
	ENSMUSG00000026712	Mrc1	5.06	7.68E-03	-5.03	4.72E-03
	ENSMUSG00000025479	Cyp2e1	5.05	9.16E-01	-5.03	9.40E-01
	ENSMUSG00000025488	Cox8b	5.24	2.67E-01	-5.21	2.63E-01
	ENSMUSG00000030278	Cidec	5.29	2.45E-01	-5.27	2.47E-01
	ENSMUSG00000061780	Cfd	5.47	7.05E-01	-5.47	7.11E-01
	ENSMUSG00000018927	Ccl6	5.53	2.74E-03	-5.51	1.76E-03
	ENSMUSG00000029373	Pf4	5.57	8.48E-03	-5.55	6.19E-03
	ENSMUSG00000074218	Cox7a1	5.6	6.43E-01	-5.57	6.79E-01
	ENSMUSG00000079419	Ms4a6c	5.61	7.68E-03	-5.58	4.82E-03
	ENSMUSG00000022878	Adipoq	5.85	1.72E-01	-5.83	1.74E-01
	ENSMUSG00000030747	Dgat2	5.87	2.25E-01	-5.84	2.16E-01
	ENSMUSG00000027559	Car3	5.97	3.11E-01	-5.94	3.16E-01

	ENSMUSG00000029304	Spp1	5.57	1.00E+00	-6.06	1.00E+00
	ENSMUSG00000040564	Apoc1	6.77	1.00E+00	-6.74	1.00E+00
	ENSMUSG00000031722	Hp	6.81	2.37E-01	-6.79	2.43E-01

Supplementary table 2: List of genes in macrophages expressing Ntn1
List of transcript levels in macrophage clusters characterized as Ntn1 high (transcript copy number > 2) compared to Ntn1 low macrophage clusters (transcript copy number < 2). Data are expressed as log_2 fold change.
